# Access to SNAP-Authorized Retailers and Diet Quality Among SNAP Recipients

**DOI:** 10.1001/jamahealthforum.2025.0677

**Published:** 2025-04-18

**Authors:** Qingxiao Li, Shuoli Zhao

**Affiliations:** 1 Department of Agricultural Economics & Agribusiness, Louisiana State University and LSU AgCenter, Baton Rouge; 2 Department of Agricultural Economics, University of Kentucky, Lexington

## Abstract

**Importance:**

Poor food environments are known to affect diet quality and health outcomes. In the US, the Supplemental Nutrition Assistance Program (SNAP) serves as a critical nutrition safety-net for millions of low-income residents; however, whether there is an association between access to SNAP-authorized retailers and diet quality among SNAP recipients remains unclear.

**Objective:**

To evaluate the association of SNAP-authorized retailer access with diet quality among SNAP recipients.

**Design, Setting, and Participants:**

This was a serial cross-sectional study using 24-hour dietary recall data from a nationally representative sample of SNAP-participating adults from 6 cycles of the restricted-use and public-access National Health and Nutrition Examination Survey from 2007 through 2008 to 2017 through 2018. Participants’ residential locations were linked to SNAP-authorized retailers at the coordinate-date level using the Historical SNAP Retailer Locator dataset. Generalized linear models were used that accounted for the complex survey design and adjusted for age, sex, race and ethnicity, education, and family poverty income ratio. Data analyses were performed from February to October 2024.

**Exposures:**

Availability of SNAP-authorized retailers within 1 mile (5 increments: ≤0.10, >0.10-0.25, >0.25-0.50, >0.50-1.00, and >1.00 mile) from the SNAP recipient’s residence, and the distance to the nearest SNAP-authorized retailer.

**Main Outcomes and Measures:**

Daily dietary quality assessed using the Healthy Eating Index-2015 (HEI-2015), a validated measure of adherence to the *2015-2020 Dietary Guidelines for Americans*. HEI-2015 scores range from 0 (no adherence) to 100 (full adherence) to the guidelines.

**Results:**

The study analysis included 5041 participants (mean age, 43.0 years; [weighted] 58% female and 42% male), with a mean (SD) HEI-2015 score of 46.88 (25.46). Residing closer to any SNAP-authorized retailer was associated with a 3.50 higher HEI-2015 (95% CI, 1.56-5.44) for those living within 0.10 miles, and 3.50 higher (95% CI, 1.46-5.55) for those living more than 0.10 to 0.25 miles compared to those living more than 1.00 miles away. Availability of superstores and grocery stores showed a positive association, with HEI-2015 scores of 7.09 (95% CI, 3.51-11.11) and 5.21 (95% CI, 3.12-7.29) higher for those within 0.10 miles, respectively. Each additional mile from any SNAP-authorized store was associated with a HEI-2015 score decrease of 0.99 (95% CI, 0.42-1.57). Subgroup analyses showed significant associations between store access and diet quality among participants with normal weight and prediabetes, whereas associations were not statistically significant for those with diabetes.

**Conclusions and Relevance:**

This cross-sectional study found that access to SNAP-authorized retailers was associated with better diet quality among SNAP recipients, with the most substantial differences observed for proximity to superstores and grocery stores. These findings highlight the potential impact of food access on diet quality, and suggest that improving the availability of SNAP-authorized retailers in low-access areas may be an important strategy for enhancing public health and reducing diet-related conditions.

## Key Points

**Question:**

Is there an association between access to Supplemental Nutrition Assistance Program (SNAP)–authorized retail stores and diet quality among SNAP recipients?

**Finding:**

This cross-sectional study, including 5041 SNAP recipients, found that living closer to SNAP-authorized retailers, particularly superstores and grocery stores, was associated with higher Healthy Eating Index-2015 scores among participants.

**Meaning:**

This finding suggests that improving access to SNAP-authorized retailers is important for supporting better diet quality among SNAP recipients in underserved areas.

## Teaser

This cross-sectional study evaluates the association of Supplemental Nutrition Assistance Program−authorized retailer access with diet quality among program recipients.

## Introduction

The Supplemental Nutrition Assistance Program (SNAP), the largest federal nutrition assistance program in the US, serves more than 40 million residents with household incomes at or below 130% of the federal poverty level through more than 250 000 SNAP-authorized retailers.^1,2,3^ However, despite this broad reach, poor dietary quality, which is associated with increased mortality risk, remains prevalent among low-income populations in the US.^4,5,6^ Although not all food retailers are authorized to accept SNAP benefits, those that are authorized are critical infrastructure through which recipients—who rely heavily on these benefits for food purchases—access food.^7,8,9^

Food environments have been associated with mortality from cancer, heart failure, and cardiorenal diseases,^10,11,12^ as well as obesity and diabetes.^13,14^ While the retail landscape has shifted toward supercenters and convenience stores that particularly affect access patterns in low-income areas,^15,16,17^ the relationship between SNAP retailer access and participants’ dietary quality remains unexamined at the national level.^8,18^

Using restricted-use National Health and Nutrition Examination Survey (NHANES) data linked with Historical SNAP Retailer Locator dataset,^3,19^ we assessed various dimensions of retail access, including both availability of and distance to different store types, while considering SNAP recipients’ health conditions. Extending beyond previous studies of general food access,^20,21^ this SNAP-specific analysis provides evidence to inform priorities and policies regarding the improvement of dietary quality and reducing diet-related health disparities among participating low-income and vulnerable populations.^22,23,24^

## Methods

The study protocol was approved by the US National Center for Health Statistics (NCHS) Research Data Center and was deemed to be exempt by the University of Kentucky Institutional Review Board. Informed consent was obtained at the time of data collection by NHANES. Informed consent was also obtained verbally during the household interview and in writing at the mobile examination center. We conducted and reported this study in accordance with the Strengthening the Reporting of Observational Studies in Epidemiology (STROBE) reporting guideline.

### Study Design and Data Sources

This serial cross-sectional study used NHANES data from 6 waves of 2-year cycles between 2007 through 2008 and 2017 through 2018 to assess associations between SNAP retailer access and diet quality among program recipients. NHANES is conducted by NCHS to collect comprehensive health and nutrition data from the noninstitutionalized US civilian population through in-person interviews and standardized physical examinations at mobile examination centers.^19^ The survey uses a complex, multistage probability sampling design to ensure representativeness.^25^In addition to the public-access NHANES data, we accessed restricted-use data files through the Research Data Center after NCHS approval.^26^ These files provided important information, such as geocoded participant locations and exact dates of interviews, examinations, dietary recalls, and most recent SNAP receipt. Before accessing the data in the Research Data Center, all individual-level information was removed by NCHS after merging with the store location dataset.

The store location data were obtained from the Historical SNAP Retailer Locator dataset, a comprehensive repository of SNAP-authorized retail food stores, maintained by the US Department of Agriculture (USDA) Food and Nutrition Services.^3^ This dataset includes retailer name, type, address, geographic coordinates, and authorization dates for retailers authorized since 1990. Integration of SNAP retailer coordinates with restricted-use NHANES data enabled precise and valid calculation of recipients’ access to authorized stores. Additional details on the SNAP retailer authorization process, data update frequency, handling of large chains, and validation efforts are provided in Supplement 1.

### Study Population

Eligible participants were adults (≥18 years), concurrently receiving SNAP benefits, and demonstrating reliable day-1 dietary recall.^27^ Reliable dietary recall was defined as the completion of the first 4 steps of the automated multiple-pass method, identification of foods and beverages consumed for each reported eating occasion, and the presence of values for all relevant variables associated with the 24-hour dietary recall.^27^ Participants who received SNAP benefits within the monthly SNAP cycle to the physical examination were considered to be SNAP recipients, with the time period adjusted based on the actual number of days in the specific month of their examination using the restricted-use NHANES data. This approach is more precise than relying on a uniform 30-day window or self-reported data.^28,29,30,31,32,33^

A total of 5762 adult SNAP recipients were surveyed across 6 NHANES data cycles (2007-2018). After excluding 381 with missing income and 368 missing education data, the analytic sample included 5041 individuals with valid day-1 dietary recall and complete covariate information. Demographic information, including race and ethnicity, was self-reported.

### Measurement of Retailer Access

Access to SNAP-authorized retailers was characterized by the availability and proximity of stores corresponding to participants’ residences. We merged restricted-use NHANES data with the Historical SNAP Retailer Locator Data to determine participants’ residential coordinates on the date of their NHANES examination, when day 1 dietary recall data information was collected. Then these coordinates were used to calculate the minimum distance to the nearest SNAP-authorized retailer, considering only retailers with active SNAP authorization on that date.

Access to SNAP participating retailers was categorized into 5 distance categories: 0.10 or closer; more than 0.10 to 0.25; more than 0.25 to 0.50; more than 0.50 to 1.0; and more than 1.00 miles. These categories were based on the distribution of store availability in the NHANES data (ranging from 12.6% to 33.7%). According to the USDA Economic Research Service Food Access Research Atlas classification,^34^ retailers were categorized into 5 types: grocery stores, supermarkets, superstores, convenience stores, and other stores (including combination and specialty stores). We evaluated associations between diet quality and both distance to and availability of each store type.

### Outcome Measures 

Diet quality was assessed using the Healthy Eating Index-2015 (HEI-2015), a validated measure of adherence to the *2015-2020 Dietary Guidelines for Americans*.^35^ The USDA Food Patterns Equivalents Database and MyPyramid Equivalents Database were used to convert dietary intake into food patterns equivalent component quantities.^36,37^ These quantities were converted to 13 HEI-2015 component scores based on the HEI-2015 scoring algorithm, including 9 adequacy components (eg, total fruits and vegetables) and 4 moderation components (eg, added sugar). The primary outcome of interest was the total HEI-2015 for the 24-hour recall, calculated by summing all component scores. HEI-2015 total scores range from 0 to 100, with higher scores indicating healthier diets more closely aligned with the *2015-2020 Dietary Guidelines for Americans* (eTable 1 in Supplement 1).

### Population Subgroups

Given recent evidence linking poor food environments to obesity-related cancer mortality and the rising burden of diabetes in the US,^38,39^ subsample analyses were conducted using NHANES data on body mass index (BMI) and hemoglobin A_1c_ (HbA_1c_) levels. BMI is a common indicator of overall adiposity and health risk, which is calculated from weight (in kilograms) divided by height (in meters squared). For this analysis, obesity status was categorized according to the World Health Organization definitions of BMI: normal (<25), overweight (25 to <30), and obesity (≥30).^40^ Additionally, laboratory data on HbA_1c_ levels, obtained from blood samples drawn during physical examinations at the mobile examination centers, were assessed. HbA_1c_ reflects the percentage of glycated hemoglobin molecules, providing a reliable estimate of average blood glucose levels during the preceding 2 to 3 months. NHANES standardized HbA_1c_ measurements using the Diabetes Control and Complications Trial reference method and performed periodic calibration to ensure consistency and accuracy. Following the US Centers for Disease Control and Prevention recommendations, we used HbA_1c_ levels to categorize diabetes status among SNAP recipients as follows: without diabetes (<5.7%), prediabetes (5.7%-6.4%), and diabetes (≥6.5%).^41^ Summary statistics for adult SNAP recipients in NHANES by weight status and diabetes status are shown in eTables 2 in Supplement 1.

### Statistical Analysis

Statistical analyses were performed from February to October 2024 using Stata, release 16.1 (StataCorp), and with statistical significance defined as a 2-sided *P* value < .05. All primary and secondary models incorporated NHANES sample weights and accounted for the complex survey design, including the primary sampling units and strata, to adjust for dietary recall nonresponse and oversampling. Generalized linear models, specifying a Gaussian family with an identity link function, were used to evaluate the association between access to SNAP-authorized retailers and diet quality. Models were adjusted for individual and intertemporal factors, including age, race and ethnicity, education level, household income, and survey wave.

To further explore the association between store proximity and diet quality, stratified analyses were conducted to evaluate differences in HEI-2015 by weight status (normal weight, overweight, obesity) and diabetes status (without diabetes, prediabetes, diabetes). These analyses aimed to identify potential effect modification and assess whether the relationship between store access and diet quality varied across health status. The results of unadjusted models are shown in eFigures 1 to 4 in Supplement 1.

## Results

The analytic sample included 5041 SNAP recipients (mean age, 43.0 years; [weighted] 58% female and 42% male; 25% Black, 21% Hispanic or Latino, 47% White, and 7% individuals of any other race or ethnicity) (Table). The mean distance to the nearest SNAP-authorized store was 0.62 (95% CI, 0.53-0.71) miles, with a weighted 15% of recipients residing 0.10 miles or closer; 29% more than 0.10 to 0.25; 26% more than 0.25 to 0.50, 16% more than 0.50 to 1.00, and 14% more than 1.00 mile away. By store type (eTable 3 in Supplement 1), the mean minimum distance was more than 2.00 miles for grocery stores (2.24 miles; 95% CI, 1.90-2.58), supermarkets (2.39 miles [95% CI, 1.95-2.83]), and superstores (2.71 miles; 95% CI, 2.32-3.10), whereas convenience stores were the closest at 0.87 miles (95% CI, 0.77-0.97). The characteristics of SNAP recipients were similar across distance bins (Table), with primary differences observed for race and ethnicity. Meanwhile, the HEI-2015 dropped slightly, from 47.93 (95% CI, 46.19-49.68) to 44.80 (95% CI, 43.89-45.71), for those who lived within 0.10 mile of a SNAP store to greater than 1.00 mile.

**Table.** Characteristics of Adult SNAP Recipients in NHANES by Their Distance to the Closest SNAP-Authorized Store

Characteristic	Weighted, mean (95% CI)^a^					
	All SNAP recipients	Distance to closest SNAP-authorized store, miles				
		≤0.10	>0.10 to 0.25	>0.25 to 0.50	>0.50 to 1.00	>1.00
SNAP recipients, No.	5041	831	1498	1331	745	636
HEI-2015 score	46.88 (46.18-47.58)	47.95 (46.55-49.34)	46.62 (45.37-47.87)	46.29 (44.37-48.21)	46.29 (44.37-48.21)	44.80 (43.89-45.71)
Total vegetables	2.66 (2.58-2.75)	2.69 (2.47-2.90)	2.74 (2.62-2.86)	2.58 (2.46-2.70)	2.60 (2.36-2.85)	2.71 (2.52-2.90)
Greens and beans	1.22 (1.10-1.34)	1.22 (0.98-1.47)	1.29 (1.11-1.48)	1.21 (1.03-1.38)	1.24 (0.99-1.49)	1.10 (0.89-1.32)
Total fruits	1.61 (1.52-1.70)	1.89 (1.60-2.18)	1.68 (1.54-1.83)	1.71 (1.59-1.84)	1.41 (1.22-1.61)	1.22 (1.06-1.37)
Whole fruits	1.43 (1.32-1.54)	1.63 (1.39-1.87)	1.46 (1.31-1.61)	1.56 (1.43-1.70)	1.31 (1.15-1.47)	1.09 (0.90-1.28)
Whole grains	1.92 (1.78-2.06)	2.04 (1.74-2.34)	1.94 (1.65-2.23)	1.83 (1.56-2.10)	2.14 (1.74-2.53)	1.67 (1.43-1.92)
Total dairy	4.78 (4.58-4.99)	4.59 (4.13-5.05)	4.76 (4.42-5.10)	4.86 (4.47-5.24)	4.78 (4.37-5.18)	4.92 (4.55-5.28)
Total protein food	4.10 (4.05-4.14)	4.17 (4.03-4.31)	4.16 (4.07-4.25)	4.06 (3.94-4.19)	4.03 (3.88-4.18)	4.02 (3.83-4.21)
Seafood and plant proteins	1.88 (1.78-1.98)	1.92 (1.70-2.14)	2.07 (1.79-2.34)	1.79 (1.60-1.99)	1.79 (1.45-2.12)	1.71 (1.49-1.94)
Fatty acids	4.78 (4.62-4.94)	4.92 (4.49-5.35)	5.00 (4.63-5.38)	4.74 (4.30-5.18)	4.55 (4.04-5.06)	4.49 (4.00-4.99)
Sodium	4.74 (4.63-4.85)	4.68 (4.39-4.98)	4.83 (4.64-5.02)	4.68 (4.36-5.01)	4.79 (4.51-5.07)	4.69 (4.46-4.92)
Refined grains	6.07 (5.92-6.23)	6.06 (5.75-6.37)	6.01 (5.68-6.34)	6.05 (5.73-6.36)	6.04 (5.58-6.50)	6.29 (5.88-6.70)
Saturated fat	6.16 (6.02-6.31)	6.28 (5.93-6.63)	6.30 (5.97-6.62)	6.08 (5.68-6.48)	6.18 (5.71-6.64)	5.92 (5.54-6.31)
Added sugars	5.52 (5.30-5.73)	5.85 (5.32-6.38)	5.71 (5.43-5.99)	5.48 (5.07-5.89)	5.45 (4.98-5.92)	4.97 (4.50-5.43)
Age, y	43.00 (42.25-43.75)	43.67 (42.32-45.02)	43.14 (41.95-44.33)	42.57 (40.88-44.26)	41.95 (40.62-43.29)	43.98 (42.11-45.84)
Female	0.58 (0.56-0.60)	0.61 (0.58-0.65)	0.56 (0.53-0.60)	0.58 (0.55-0.61)	0.57 (0.52-0.62)	0.58 (0.53-0.62)
Male	0.42 (0.40-0.44)	0.39 (0.35-0.42)	0.44 (0.40-0.47)	0.42 (0.39-0.45)	0.43 (0.38-0.48)	0.42 (0.38-0.47)
Race and ethnicity						
Black	0.25 (0.21-0.29)	0.30 (0.24-0.36)	0.32 (0.26-0.37)	0.25 (0.20-0.30)	0.20 (0.16-0.24)	0.08 (0.02-0.14)
Hispanic	0.21 (0.18-0.25)	0.31 (0.22-0.40)	0.23 (0.17-0.29)	0.22 (0.16-0.27)	0.17 (0.11-0.24)	0.11 (0.06-0.16)
White	0.47 (0.43-0.52)	0.31 (0.22-0.39)	0.37 (0.32-0.42)	0.47 (0.42-0.53)	0.57 (0.48-0.65)	0.75 (0.68-0.82)
Other^b^	0.07 (0.06-0.09)	0.09 (0.04-0.13)	0.08 (0.05-0.11)	0.06 (0.04-0.07)	0.06 (0.03-0.08)	0.06 (0.02-0.10)
Education level						
<High school	0.34 (0.31-0.36)	0.37 (0.31-0.43)	0.34 (0.30-0.38)	0.33 (0.29-0.37)	0.27 (0.21-0.34)	0.36 (0.30-0.41)
High school	0.31 (0.29-0.33)	0.28 (0.24-0.32)	0.30 (0.26-0.35)	0.32 (0.28-0.37)	0.37 (0.30-0.43)	0.28 (0.24-0.32)
Some college	0.29 (0.27-0.31)	0.30 (0.25-0.35)	0.29 (0.26-0.32)	0.28 (0.25-0.31)	0.30 (0.25-0.36)	0.31 (0.25-0.38)
College	0.06 (0.05-0.08)	0.05 (0.04-0.07)	0.07 (0.05-0.09)	0.06 (0.03-0.10)	0.06 (0.03-0.09)	0.05 (0.03-0.07)
Poverty ratio^c^						
<1.30	0.72 (0.69-0.74)	0.75 (0.67-0.83)	0.71 (0.66-0.76)	0.73 (0.68-0.77)	0.69 (0.61-0.77)	0.69 (0.63-0.75)
1.30 to <1.85	0.13 (0.11-0.15)	0.11 (0.08-0.14)	0.12 (0.09-0.16)	0.14 (0.11-0.17)	0.13 (0.07-0.18)	0.15 (0.10-0.20)
1.85 to <3.00	0.10 (0.08-0.12)	0.09 (0.03-0.16)	0.10 (0.06-0.15)	0.09 (0.05-0.13)	0.13 (0.07-0.18)	0.09 (0.05-0.14)
>3.00	0.06 (0.04-0.07)	0.05 (0.00-0.09)	0.06 (0.03-0.09)	0.05 (0.01-0.09)	0.06 (0.02-0.10)	0.06 (0.02-0.11)
Survey wave						
2007-2008	0.12 (0.08-0.15)	0.10 (0.06-0.14)	0.11 (0.07-0.16)	0.12 (0.07-0.16)	0.11 (0.04-0.17)	0.17 (0.06-0.27)
2009-2010	0.15 (0.12-0.18)	0.15 (0.08-0.22)	0.17 (0.11-0.22)	0.14 (0.10-0.19)	0.09 (0.03-0.15)	0.20 (0.09-0.30)
2011-2012	0.19 (0.14-0.23)	0.20 (0.09-0.31)	0.23 (0.16-0.30)	0.16 (0.10-0.22)	0.18 (0.10-0.26)	0.16 (0.07-0.25)
2013-2014	0.19 (0.15-0.23)	0.16 (0.08-0.25)	0.18 (0.11-0.25)	0.22 (0.17-0.28)	0.20 (0.12-0.28)	0.18 (0.09-0.27)
2015-2016	0.18 (0.14-0.22)	0.19 (0.12-0.26)	0.15 (0.11-0.19)	0.20 (0.13-0.27)	0.20 (0.11-0.29)	0.16 (0.05-0.27)
2017-2018	0.17 (0.15-0.20)	0.20 (0.09-0.30)	0.17 (0.12-0.21)	0.16 (0.11-0.20)	0.22 (0.14-0.31)	0.14 (0.06-0.23)

Abbreviations: HEI, Healthy Eating Index-15; NHANES, US National Health and Nutrition Examination Survey; SNAP, Supplemental Nutrition Assistance Program.

^a^ Mean values were adjusted for NHANES dietary weights. These weights account for the complex survey design, including oversampling, survey nonresponse, and poststratification.

^b^ Any other self-reported race and/or ethnicity, including multiracial.

^c^ Represents the ratio of family income to the federal poverty threshold, adjusted for household size. Higher ratios indicate higher income levels.

### Availability of SNAP-Authorized Store and Dietary Quality

**Figure 1.**
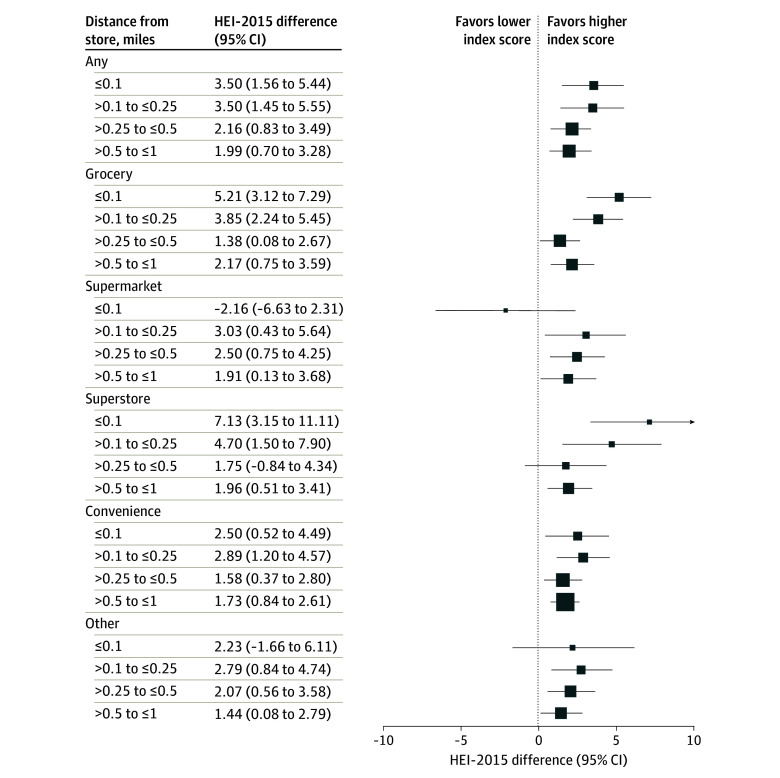
Estimated Healthy Eating Index-15 (HEI-2015) Difference by Availability of Supplemental Nutrition Assistance Program (SNAP)–Authorized Store Types Among Recipients Residing More Than 1 Mile Away Squares represent the estimated HEI-2015 differences for SNAP recipients by availability of various SNAP-authorized store types within specified distances relative to being more than 1 mile away, with horizontal lines indicating the 95% CIs. The store types included were grocery stores, supermarkets, superstores, and convenience stores, as well as a category for all store types combined. Positive coefficients indicate higher HEI-2015 for closer proximity to the respective store type compared to being farther than 1 mile away.

Figure 1 shows the estimated association of SNAP-authorized store availability with HEI-2015 among SNAP recipients for the composite category “any” (all store types combined), all distances within 1.00 mile were significantly associated with higher scores compared to living more than 1.00 mile away. Specifically, the HEI-2015 was 3.50 higher (95% CI, 1.56-5.44) for SNAP recipients living within 0.1 miles of any SNAP-authorized store compared with those living beyond 1 mile. A similar association (3.50 higher; 95% CI, 1.45-5.55) was observed for those living 0.10 to 0.25 mile away.

Among individual store types, convenience stores replicated this pattern, with all distances less than 1.00 mile showing statistically significant associations (range, 1.58-2.89). Superstore availability also showed statistically significant associations, with HEI-2015 of 7.13 higher (95% CI, 3.51-11.11) for participants within 0.10 miles and 4.70 higher (95% CI, 1.50-7.90) for those 0.10 to 0.25 miles away. For grocery stores, HEI-2015 was 5.21 higher (95% CI, 3.12-7.29) within 0.10 miles and 3.85 higher (95% CI, 2.24-5.45) at 0.10 to 0.25 miles. For supermarkets, availability was associated with HEI-2015 3.03 higher (95% CI, 0.43-5.64) at 0.10 to 0.25 miles and 2.50 higher (95% CI, 0.75-4.25) at 0.25 to 0.50 miles. Although most SNAP stores showed positive associations at closer distances, some of the shortest distances, such as supermarkets within 0.10 miles, yielded negative but nonstatistically significant estimates (−2.16; 95% CI, −6.63 to 2.31). This result is likely attributable to the limited sample size of SNAP recipients residing within 0.10 miles of a SNAP-authorized supermarket (eTable 3 in Supplement 1).

### Availability of SNAP-Authorized Stores and Dietary Quality by Weight Status

**Figure 2.**
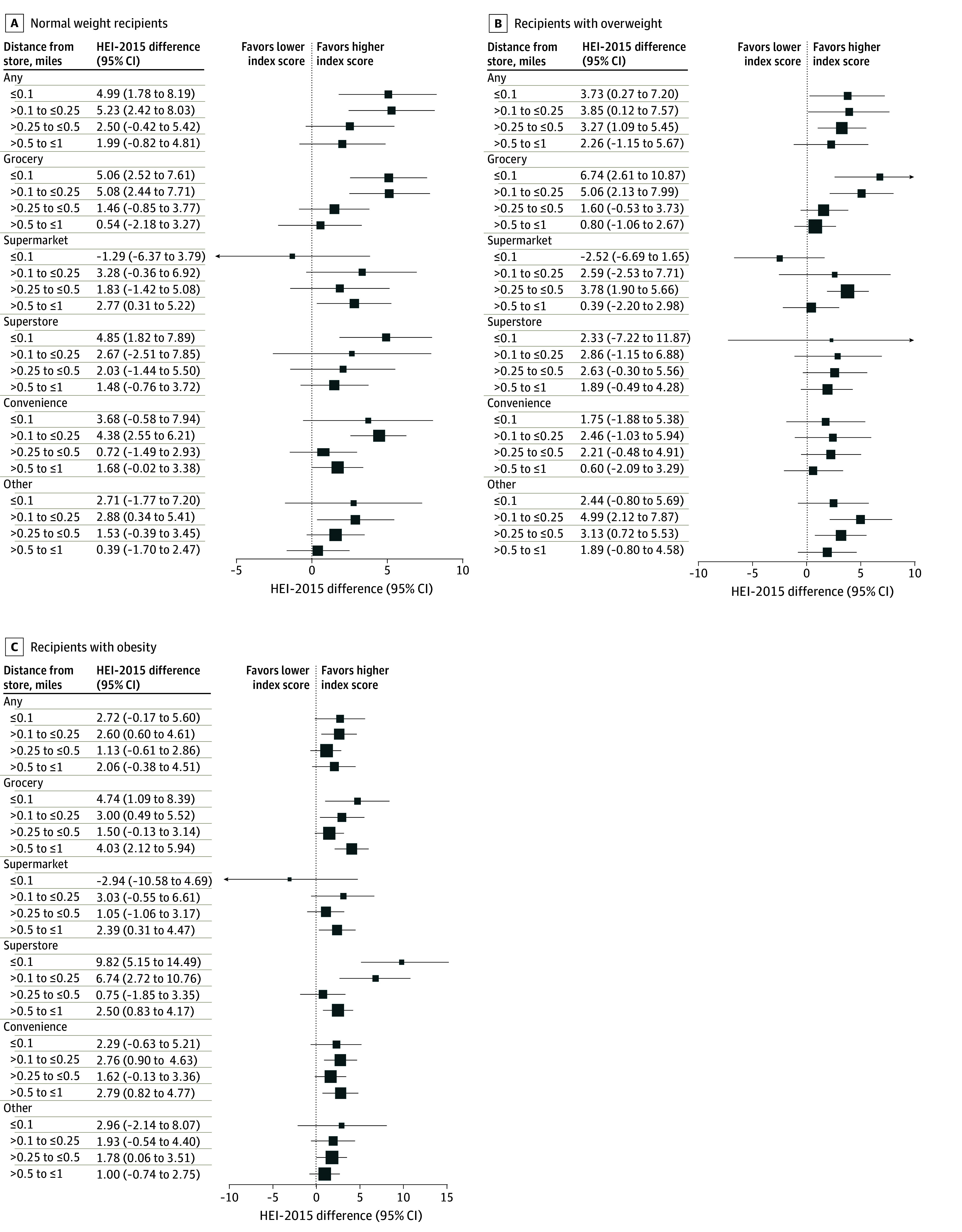
Estimated Healthy Eating Index-2015 (HEI-2015) Difference by Availability of Supplemental Nutrition Assistance Program (SNAP)–Authorized Store Types Among Recipients by Weight Status, Relative to Living More Than 1 Mile Away Squares represent the estimated HEI-2015 differences for SNAP recipients by availability of SNAP-authorized store types at specified distances.

Figure 2 shows the estimated differences in HEI-2015 by weight status. Among SNAP recipients with normal weight (BMI <25), the HEI-2015 was 4.99 (95% CI, 1.78-8.19) higher within 0.10 miles of any SNAP-authorized store and 5.23 (95% CI, 2.42-8.03) higher at 0.10 to 0.25 miles, compared with those farther than 1.00 mile. When evaluated by specific store types, grocery store availability at 0.10 to 0.25 miles was associated with HEI-2015 of 5.08 higher (95% CI, 2.44-7.71), and within 0.10 mile with HEI-2015 of 5.06 higher (95% CI, 2.52-7.61). Among participants with overweight (BMI ≥25), the HEI-2015 was 3.73 (95% CI, 0.27-7.20) higher within 0.10 mile of any store. The most pronounced differences were observed for grocery store availability, with HEI-2015 of 6.73 higher (95% CI, 2.61-10.87) within 0.10 miles and 5.06 higher (95% CI, 2.13-7.99) at 0.10 to 0.25 miles. For participants with obesity participants (BMI ≥30), the greatest differences were observed for superstore availability, with 9.82 higher (95% CI, 5.15-14.49) in HEI-2015 for SNAP recipients living within 0.10 miles and 6.74 higher (95% CI, 2.72-10.76) at 0.10 to 0.25 miles. Grocery store availability within 0.1 miles was associated with HEI-2015 of 4.74 higher (95% CI, 1.09-8.39).

### Availability of SNAP-Authorized Stores and Dietary Quality by Diabetes Status

**Figure 3.**
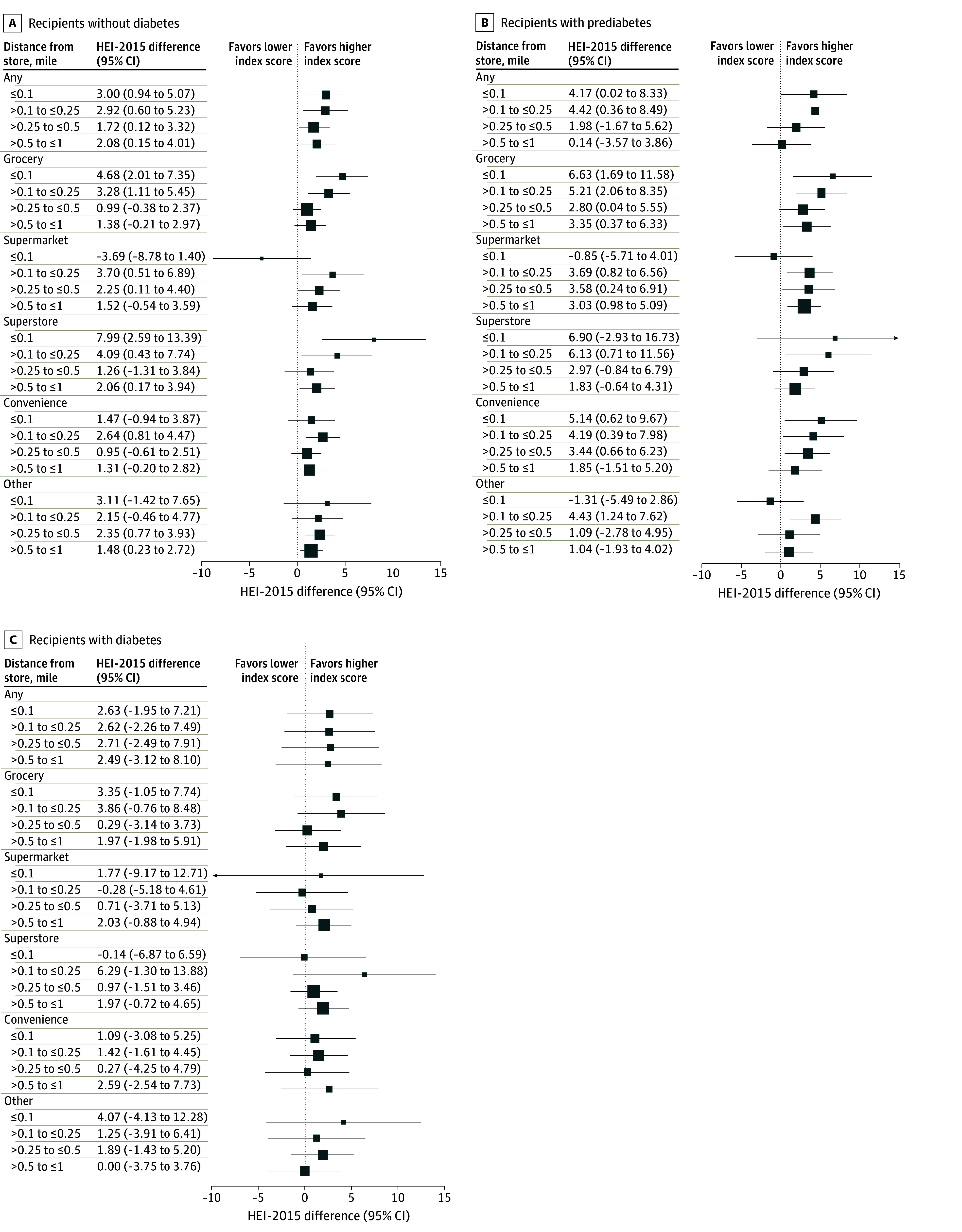
Estimated Healthy Eating Index-2015 (HEI-2015) Difference by Availability of Supplemental Nutrition Assistance Program (SNAP)–Authorized Store Types Among SNAP Recipients by Diabetes Status, Relative to Living More Than 1 Mile Away Squares represent the estimated HEI-2015 differences for SNAP recipients by availability of SNAP-authorized store types at specified distances.

Figure 3 shows the estimated differences in HEI-2015 by diabetes status. Among participants without diabetes, the HEI-2015 was 3.00 higher (95% CI, 0.94-5.07) within 0.10 miles of any store compared with those beyond 1.00 mile, with consistent differences observed at 0.10 to 0.25 miles (2.92 higher; 95% CI, 0.60-5.23), 0.25 to 0.50 miles (1.72 higher; 95% CI, 0.12-3.32), and 0.50 to 1.00 mile (2.08 higher; 95% CI, 0.15-4.01). When evaluated by specific store types, superstore availability showed the strongest associations, with HEI-2015 of 7.99 higher (95% CI, 2.59-13.39) within 0.10 miles and 4.09 higher (95% CI, 0.43-7.74) at 0.10 to 0.25 miles. Grocery store availability was associated with HEI-2015 of 4.68 higher (95% CI, 2.01-7.35) within 0.10 miles and 3.28 higher (95% CI, 1.11-5.45) at 0.10 to 0.25 miles.

Among participants with prediabetes, the HEI-2015 was 4.17 higher (95% CI, 0.02-8.33) within 0.10 miles of any SNAP-authorized store and 4.42 higher (95% CI, 0.36-8.49) at 0.10 to 0.25 miles. Grocery store availability showed statistically significant associations, with HEI-2015 of 6.63 higher (95% CI, 1.69-11.58) within 0.1 miles and 5.21 higher (95% CI, 2.06-8.35) at 0.10 to 0.25 miles. Lastly, we observed positive but insignificant associations among participants with diabetes, regardless of store availability or store type.

### Distance to Nearest SNAP-Authorized Store and Dietary Quality 

**Figure 4.**
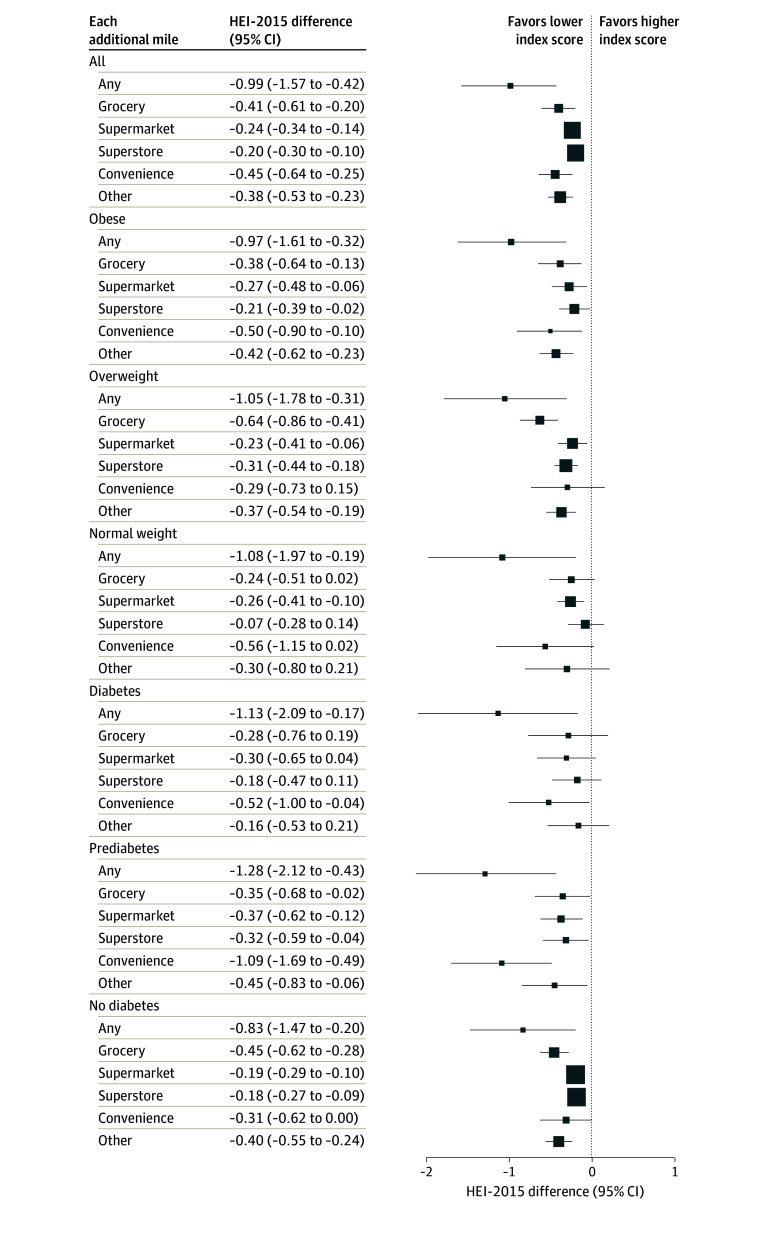
Estimated Healthy Eating Index-2015 (HEI-2015) Difference by Distance to Supplemental Nutrition Assistance Program (SNAP)–Authorized Store Types, by Obesity and Diabetes Status Squares represent the estimated differences in HEI-2015 scores per additional mile of distance to various SNAP-authorized store types.

Figure 4 shows the estimated association between distance to the nearest SNAP-authorized store and HEI-2015 among SNAP recipients, stratified by weight status and diabetes status. Each additional mile that SNAP recipients lived from any SNAP-authorized store was associated with a decrease of 0.99 (95% CI, 0.42 to 1.57) in HEI-2015. The largest decrease in dietary quality was associated with distance to convenience stores (−0.45; 95% CI, −0.64 to −0.25), followed by grocery stores (−0.41; 95% CI, −0.61 to −0.20). Supermarkets and superstores showed smaller but still significant associations, with decreases of 0.24 (95% CI, 0.14 to 0.34) and 0.20 (95% CI, 0.10 to 0.30) in HEI-2015 per additional mile, respectively.

Weight and diabetes status stratification showed consistent negative associations across subgroups. Participants with obesity, overweight, and normal weight experienced decreases in HEI-2015 of 0.97 (95% CI, 0.32 to 1.61), 1.05 (95% CI, 0.31 to 1.78), and 1.08 (95% CI, 0.19 to 1.97) per additional mile from any SNAP-authorized store, respectively. HEI-2015 was also associated with decreases of 1.13 (95% CI, 0.17 to 2.09), 1.28 (95% CI, 0.43 to 2.12), and 0.83 (95% CI, 0.20 to 1.47) per additional mile from SNAP-authorized store for participants with diabetes, prediabetes, and without diabetes. Convenience stores maintained the statistically significant negative associations across weight and diabetes categories, whereas grocery stores also showed substantial effects, particularly for participants with overweight (−0.64; 95% CI, −0.86 to −0.41).

## Discussion

In this serial cross-sectional study of SNAP recipients, we observed higher dietary quality among those with better access to SNAP-authorized stores, particularly within 0.25 miles of superstores and grocery stores. This finding aligns with previous research demonstrating the positive connection between accessing superstores and grocery stores on diet quality.^42^ These 2 types of stores typically offer a wider variety of fresh produce, whole grains, and lean proteins, facilitating healthier food choices.^43^ Additionally, in contrast with previous literature,^44^ the availability of convenience stores within short distances showed a positive association with HEI-2015.

We also found a significant inverse relationship between the minimum distance to SNAP-authorized stores and diet quality of SNAP recipients. This finding aligns with existing research indicating that increased distance to food retailers can negatively affect diet quality because it may limit access to and consumption of nutritious foods.^45^ The negative association was observed across all store types, with convenience stores exhibiting the largest association magnitude. This may be because convenience stores are still the most accessible type of store among SNAP recipients.^46,47^

Furthermore, we evaluated the associations between store proximity and diet quality in subsamples of SNAP recipients based on weight status and diabetes status. Specifically, obese SNAP recipients showed weak relationships between closer store availability and improved dietary quality. Studies have shown that even with access to healthy options, individuals with obesity may encounter challenges with dietary adherence due to ingrained eating habits, limited nutrition knowledge, and the pervasive marketing of unhealthy foods.^48,49,50^ These observations suggest that improving access alone may not fully address the dietary quality of individuals with obesity. Meanwhile, the association between SNAP-authorized store access and dietary quality was statistically insignificant among participants with diabetes, likely due to unique barriers, such as the need for specialized dietary guidance that is difficult to follow on a limited budget.^39,51,52,53^ Psychological and social factors following a diabetes diagnosis, such as stress, depression, and social isolation, can impact motivation, self-efficacy, and the ability to sustain healthy choices.^54^ Therefore, addressing the dietary needs of SNAP recipients with health conditions may require a comprehensive approach beyond improving their access to stores.

Prior studies have shown that food environments can influence dietary behaviors and health outcomes.^10,23,42,55,56,57,58,59,60^ Our study extends this evidence by examining SNAP recipients, a population representing 22 million families who rely on authorized retailers for most of their food purchases,^61,62^ and may face consequential health risks associated with food access and diet quality.^32,63^

Our results highlight the need for policies aimed at improving the SNAP retail environment for these participants.^64^ Increasing the density of SNAP-authorized retailers in underserved areas may improve access to healthy and affordable foods for SNAP families. Policy interventions could include incentives such as tax breaks, zoning adjustments, and grant programs to encourage the establishment of new stores.

Over the decades, SNAP policy has evolved substantially, evolving from a food-stamp program to a comprehensive electronic benefit transfer (EBT) system. Recent policy discussions have prioritized expanding online EBT options and refining eligibility criteria for SNAP-authorized retailers.^65,66^ Our findings support these efforts, highlighting the importance of improving access to healthy food and the potential cost-effectiveness of expanding online EBT. Additionally, re-evaluating retailer eligibility requirements could promote a broader range of authorized retailers in underserved areas by streamlining the authorization process, updating standards, and assisting smaller stores in meeting the necessary standards.

### Strengths and Limitations

The strengths of this study include the use of restricted NHANES data-enabled precise linkage of individual dietary records with store locations through coordinate and intertemporal identifiers, thereby facilitating a detailed assessment of how access to SNAP-authorized stores affects the diet quality of SNAP recipients. Additionally, our findings regarding store proximity and dietary quality among SNAP recipients with different weights and diabetes status provide insight into these relationships in specific populations.

This study also had several limitations. First, self-reported 24-hour dietary recalls may introduce the potential for measurement error due to day-to-day variability in food intake. Second, the exclusion of participants with incomplete socioeconomic data, including education and income, may affect generalizability. Third, while straight-line distance calculations provide standardized accessibility measures, they do not account for actual and common travel factors such as road networks, traffic patterns, physical barriers, and transportation options that could influence store access. Nevertheless, this approach is consistent with previous food access studies,^46,65^ and provides a robust measure of store accessibility. Fourth, this analysis lacked data on where SNAP recipients purchased their food, limiting our understanding of how different store types may contribute to dietary patterns.

## Conclusions

This cross-sectional study found that living closer to and having better availability of SNAP-authorized stores, particularly superstores and grocery stores, was associated with higher HEI-2015 scores among participants. This analysis further evaluated this association for different weight and diabetes status categories. These findings suggest that improving access to SNAP retailers in underserved areas may enhance dietary quality among SNAP recipients. Future research incorporating food purchasing data would help to further characterize the specific role of different SNAP-authorized retailers.

## Notes

Supplement 1. eTable 1. Healthy Eating Index-2015 scoring criteria standards eTable 2. Characteristics of Adult SNAP Participants in the NHANES by Weight and Diabetes Status eTable 3. Store Availability and Average Minimum Distance to SNAP-Authorized Stores Among Adult SNAP Participants in the NHANES eFigure 1. Unadjusted Estimated HEI-2015 Difference by Availability of SNAP-Authorized Store Types Among SNAP Recipients, Relative to Living More Than 1 Mile Away eFigure 2. Unadjusted Estimated HEI-2015 Difference by Availability of SNAP-Authorized Store Types Among SNAP Recipients by Weight Status, Relative to Living More Than 1 Mile Away eFigure 3. Unadjusted Estimated HEI-2015 Difference by Availability of SNAP-Authorized Store Types Among SNAP Recipients by Diabetes Status, Relative to Living More Than 1 Mile Away eFigure 4. Unadjusted Estimated HEI-2015 Difference per Additional Mile of Distance to Nearest SNAP-Authorized Store Types Among SNAP Recipients, by Obesity and Diabetes Status eFigure 5. Estimated HEI-2015 Component Differences per Additional Mile of Distance to SNAP-Authorized Stores Among SNAP Recipients eFigure 6. Unadjusted Estimated HEI-2015 Component Differences per Additional Mile of Distance to SNAP-Authorized Stores Among SNAP Recipients

Supplement 2. Data Sharing Statement
