# Supplementary material for: Access to SNAP-Authorized Retailers and Diet Quality Among SNAP Recipients
Source: JAMA Health Forum. 2025 Apr 18;6(4):e250677. doi: 10.1001/jamahealthforum.2025.0677 (PMC12008766; doi:10.1001/jamahealthforum.2025.0677)
Supplement: Supplement 1. — eTable 1. Healthy Eating Index-2015 scoring criteria standards eTable 2. Characteristics of Adult SNAP Participants in the NHANES by Weight and Diabetes Status eTable 3. Store Availability and Average Minimum Distance to SNAP-Authorized Stores Among Adult SNAP Participants in the NHANES eFigure 1. Unadjusted Estimated HEI-2015 Difference by Availability of SNAP-Authorized Store Types Among SNAP Recipients, Relative to Living More Than 1 Mile Away eFigure 2. Unadjusted Estimated HEI-2015 Difference by Availability of SNAP-Authorized Store Types Among SNAP Recipients by Weight Status, Relative to Living More Than 1 Mile Away eFigure 3. Unadjusted Estimated HEI-2015 Difference by Availability of SNAP-Authorized Store Types Among SNAP Recipients by Diabetes Status, Relative to Living More Than 1 Mile Away eFigure 4. Unadjusted Estimated HEI-2015 Difference per Additional Mile of Distance to Nearest SNAP-Authorized Store Types Among SNAP Recipients, by Obesity and Diabetes Status eFigure 5. Estimated HEI-2015 Component Differences per Additional Mile of Distance to SNAP-Authorized Stores Among SNAP Recipients eFigure 6. Unadjusted Estimated HEI-2015 Component Differences per Additional Mile of Distance to SNAP-Authorized Stores Among SNAP Recipients [file jamahealthforum-e250677-s001.pdf]

Supplemental Online Content

Li Q, Zhao S. Access to SNAP-Authorized Retailers and Diet Quality Among SNAP Recipients. *JAMA Health Forum*. Published online April 18, 2025. doi:10.1001/jamahealthforum.2025.0677

eTable 1. Healthy Eating Index-2015 scoring criteria standards
eTable 2. Characteristics of Adult SNAP Participants in the NHANES by Weight and Diabetes Status
eTable 3. Store Availability and Average Minimum Distance to SNAP-Authorized Stores Among Adult SNAP Participants in the NHANES
eFigure 1. Unadjusted Estimated HEI-2015 Difference by Availability of SNAP-Authorized Store Types Among SNAP Recipients, Relative to Living More Than 1 Mile Away
eFigure 2. Unadjusted Estimated HEI-2015 Difference by Availability of SNAP-Authorized Store Types Among SNAP Recipients by Weight Status, Relative to Living More Than 1 Mile Away
eFigure 3. Unadjusted Estimated HEI-2015 Difference by Availability of SNAP-Authorized Store Types Among SNAP Recipients by Diabetes Status, Relative to Living More Than 1 Mile Away
eFigure 4. Unadjusted Estimated HEI-2015 Difference per Additional Mile of Distance to Nearest SNAP-Authorized Store Types Among SNAP Recipients, by Obesity and Diabetes Status
eFigure 5. Estimated HEI-2015 Component Differences per Additional Mile of Distance to SNAP-Authorized Stores Among SNAP Recipients
eFigure 6. Unadjusted Estimated HEI-2015 Component Differences per Additional Mile of Distance to SNAP-Authorized Stores Among SNAP Recipients

This supplemental material has been provided by the authors to give readers additional information about their work.

© 2025 Li Q & Zhao S. *JAMA Health Forum*.

Supplemental Information on SNAP Retailer Locator Data

SNAP Retailer Locator Data: The USDA's Food and Nutrition Service (FNS) authorizes retailers to participate in SNAP. Retailers must apply to FNS and meet specific eligibility criteria related to the types of food they sell (e.g., staple foods). FNS reviews applications and conducts site visits before granting authorization. FNS retailer information is considered to be updated on an ongoing basis as new stores are authorized, existing stores are deauthorized, and store information changes. They provide monthly updates for researchers. The Historical SNAP Retailer Locator Data used in this study includes the dates of authorization and deauthorization for each store. This allows researchers to identify the active SNAP retailers in any given timeframe since 1990. This is the only official SNAP Retailer Locator data at the national level, which was first released in 2023.

Handling of Large Chains: For large retail chains, each individual store location must be authorized to accept SNAP benefits. While the parent company can submit a single application covering multiple locations, each store is evaluated separately to ensure compliance with SNAP requirements. This ensures that all locations meet the necessary criteria to participate in the program.

Data Validation Efforts: FNS retailer information is updated on an ongoing basis as new stores are authorized, existing stores are deauthorized, and store information changes. They provide monthly updates for researchers. The Historical SNAP Retailer Locator Data used in this study includes the dates of authorization and deauthorization for each store. This allows researchers to identify the active SNAP retailers in any given timeframe since 1990. This is the only official SNAP Retailer Locator data at the national level, which was released in 2023. (https://www.fns.usda.gov/snap/retailer/data)

© 2025 Li Q & Zhao S. JAMA Health Forum.

eTable 1: Healthy Eating Index-2015 scoring criteria standards

Component	Max Points	Scoring Criteria	Standards for Maximum Score	Standards for Minimum Score
**Total Fruits**	5	Adequacy	≥0.8 cup equivalents per 1,000 kcal	No fruits
**Whole Fruits**	5	Adequacy	≥0.4 cup equivalents per 1,000 kcal	No whole fruits
**Total Vegetables**	5	Adequacy	≥1.1 cup equivalents per 1,000 kcal	No vegetables
**Greens and Beans**	5	Adequacy	≥0.2 cup equivalents per 1,000 kcal	No dark green vegetables or legumes
**Whole Grains**	10	Adequacy	≥1.5 oz equivalents per 1,000 kcal	No whole grains
**Dairy**	10	Adequacy	≥1.3 cup equivalents per 1,000 kcal	No dairy
**Total Protein Foods**	5	Adequacy	≥2.5 oz equivalents per 1,000 kcal	No protein foods
**Seafood and Plant Proteins**	5	Adequacy	≥0.8 oz equivalents per 1,000 kcal	No seafood or plant proteins
**Fatty Acids**	10	Adequacy	Ratio of polyunsaturated and monounsaturated to saturated fats ≥2.5	Ratio of polyunsaturated and monounsaturated to saturated fats ≤1.2
**Refined Grains**	10	Moderation	≤1.8 oz equivalents per 1,000 kcal	≥4.3 oz equivalents per 1,000 kcal
**Sodium**	10	Moderation	≤1.1 grams per 1,000 kcal	≥2.0 grams per 1,000 kcal
**Added Sugars**	10	Moderation	≤6.5% of total energy intake	≥26% of total energy intake
**Saturated Fats**	10	Moderation	≤8% of total energy intake	≥16% of total energy intake

Notes:

- The HEI-2015 gives higher scores for components considered **adequacy** when the intake is high, while it gives higher scores for components classified as **moderation** when the intake is low.
- The scores for each component are prorated if the intake is between the minimum and maximum standards. For instance, a partial amount of total fruits will get a partial score between 0 and 5.
- Source: Krebs-Smith SM, Pannucci TE, Subar AF, Kirkpatrick SI, Lerman JL, Tooze JA, Wilson MM, and Reedy J. Update of the Healthy Eating Index-2015. J Acad Nutr Diet. 2018 Sep;118(9):1591-1602.

© 2025 Li Q & Zhao S. JAMA Health Forum.

eTable 2. Characteristics of Adult SNAP Participants in the NHANES by Weight and Diabetes Status

	Weighted Mean (95% CI)[a]					
	Obesity status[b]			Diabetes[c]		
Characteristic	Normal weight (n=1283)	Overweight (n=1415)	Obese (n=2285)	No diabetes (n=2834)	Pre-Diabetes (n=1333)	Diabetes (n=638)
Closest SNAP store distance, mile						
Any store type	0.67 (0.50, 0.84)	0.60 (0.50, 0.69)	0.60 (0.52, 0.69)	0.64 (0.52, 0.75)	0.57 (0.46, 0.68)	0.64 (0.52, 0.76)
Grocery	2.40 (1.86, 2.93)	2.07 (1.79, 2.35)	2.22 (1.81, 2.63)	2.25 (1.93, 2.58)	2.15 (1.73, 2.57)	2.45 (2.00, 2.90)
Supermarket	2.39 (1.84, 2.95)	2.60 (1.98, 3.22)	2.27 (1.92, 2.63)	2.47 (2.00, 2.93)	2.17 (1.72, 2.62)	2.54 (1.96, 3.12)
Superstore	2.79 (2.19, 3.39)	2.88 (2.34, 3.42)	2.58 (2.23, 2.93)	2.75 (2.31, 3.19)	2.56 (2.10, 3.02)	2.85 (2.38, 3.31)
Convenience	0.94 (0.77, 1.11)	0.87 (0.72, 1.02)	0.83 (0.72, 0.93)	0.91 (0.80, 1.03)	0.75 (0.60, 0.89)	0.91 (0.75, 1.06)
Other[d]	1.29 (1.00, 1.58)	1.46 (1.01, 1.92)	1.30 (0.88, 1.72)	1.43 (1.04, 1.81)	1.12 (0.80, 1.43)	1.40 (1.04, 1.77)
HEI-2015	47.28 (46.20, 48.36)	48.15 (47.10, 49.21)	45.87 (45.00, 46.74)	46.36 (45.43, 47.29)	47.04 (46.05, 48.03)	49.39 (48.10, 50.69)
Total vegetables	2.56 (2.40, 2.72)	2.74 (2.57, 2.90)	2.68 (2.55, 2.81)	2.62 (2.50, 2.75)	2.68 (2.52, 2.84)	2.89 (2.67, 3.12)
Greens and beans	1.26 (1.00, 1.51)	1.26 (1.07, 1.45)	1.17 (1.07, 1.27)	1.20 (1.00, 1.39)	1.28 (1.11, 1.45)	1.34 (1.14, 1.54)
Total fruits	1.58 (1.43, 1.74)	1.62 (1.48, 1.77)	1.60 (1.48, 1.72)	1.52 (1.40, 1.64)	1.73 (1.53, 1.93)	1.84 (1.61, 2.07)
Whole fruits	1.39 (1.24, 1.55)	1.55 (1.39, 1.70)	1.36 (1.24, 1.49)	1.29 (1.17, 1.42)	1.61 (1.41, 1.82)	1.84 (1.60, 2.08)
Whole grains	2.06 (1.80, 2.32)	1.95 (1.70, 2.20)	1.83 (1.65, 2.02)	1.82 (1.65, 1.99)	1.94 (1.63, 2.26)	2.37 (2.07, 2.67)
Total dairy	4.58 (4.18, 4.97)	5.02 (4.62, 5.42)	4.76 (4.51, 5.02)	4.79 (4.60, 4.98)	4.77 (4.40, 5.15)	5.01 (4.61, 5.41)
Total protein food	4.05 (3.93, 4.17)	4.13 (4.03, 4.23)	4.09 (4.00, 4.18)	4.06 (3.99, 4.14)	4.09 (3.99, 4.19)	4.27 (4.12, 4.42)
Seafood and plant proteins	1.98 (1.77, 2.20)	1.93 (1.72, 2.14)	1.79 (1.70, 1.88)	1.84 (1.69, 2.00)	1.91 (1.73, 2.09)	2.04 (1.81, 2.27)
Fatty acids	4.81 (4.57, 5.06)	4.83 (4.30, 5.36)	4.73 (4.52, 4.93)	4.70 (4.54, 4.87)	4.80 (4.53, 5.07)	4.97 (4.44, 5.49)
Sodium	4.93 (4.64, 5.22)	5.01 (4.68, 5.33)	4.49 (4.25, 4.73)	4.87 (4.69, 5.05)	4.70 (4.30, 5.10)	4.04 (3.55, 4.54)
Refined grains	6.36 (6.10, 6.63)	6.28 (6.05, 6.51)	5.82 (5.59, 6.06)	6.11 (5.96, 6.26)	6.05 (5.69, 6.41)	5.80 (5.30, 6.30)
Saturated fat	6.40 (6.15, 6.66)	6.18 (5.74, 6.63)	6.02 (5.77, 6.26)	6.16 (5.98, 6.34)	6.04 (5.72, 6.35)	6.25 (5.76, 6.75)
Added sugars	5.31 (4.99, 5.62)	5.66 (5.37, 5.96)	5.53 (5.24, 5.82)	5.37 (5.09, 5.66)	5.43 (4.94, 5.92)	6.73 (6.32, 7.15)
Age	41.63 (40.25, 43.01)	43.36 (41.95, 44.77)	43.45 (42.64, 44.25)	38.51 (37.69, 39.34)	49.01 (47.98, 50.03)	55.92 (54.69, 57.14)
Gender (male)	0.51 (0.48, 0.53)	0.47 (0.44, 0.50)	0.34 (0.31, 0.36)	0.42 (0.40, 0.44)	0.43 (0.39, 0.47)	0.42 (0.37, 0.47)
Race/ethnicity						
White	0.50 (0.44, 0.57)	0.46 (0.39, 0.52)	0.47 (0.43, 0.52)	0.50 (0.45, 0.55)	0.43 (0.37, 0.48)	0.47 (0.43, 0.52)
Black	0.25 (0.19, 0.31)	0.22 (0.17, 0.26)	0.26 (0.22, 0.30)	0.21 (0.17, 0.25)	0.30 (0.25, 0.36)	0.26 (0.21, 0.31)
Hispanic	0.14 (0.11, 0.18)	0.27 (0.21, 0.32)	0.22 (0.18, 0.26)	0.22 (0.18, 0.26)	0.20 (0.16, 0.23)	0.21 (0.17, 0.25)
Other[e]	0.11 (0.08, 0.14)	0.06 (0.04, 0.09)	0.05 (0.04, 0.07)	0.07 (0.05, 0.09)	0.07 (0.05, 0.09)	0.06 (0.04, 0.08)
Education						
Less than high school	0.33 (0.30, 0.37)	0.36 (0.32, 0.40)	0.32 (0.29, 0.35)	0.32 (0.29, 0.34)	0.34 (0.29, 0.40)	0.41 (0.36, 0.47)
High school	0.32 (0.29, 0.36)	0.32 (0.28, 0.36)	0.31 (0.28, 0.33)	0.32 (0.30, 0.35)	0.31 (0.26, 0.35)	0.26 (0.21, 0.31)
Some college	0.27 (0.24, 0.30)	0.27 (0.23, 0.31)	0.32 (0.29, 0.36)	0.30 (0.28, 0.33)	0.28 (0.24, 0.32)	0.28 (0.21, 0.34)
College	0.07 (0.04, 0.11)	0.06 (0.04, 0.08)	0.06 (0.04, 0.07)	0.06 (0.04, 0.08)	0.07 (0.04, 0.10)	0.05 (0.03, 0.07)
Poverty ratio[f]						
<1.30	0.72 (0.67, 0.76)	0.73 (0.70, 0.76)	0.71 (0.66, 0.75)	0.72 (0.68, 0.76)	0.69 (0.65, 0.73)	0.73 (0.67, 0.80)
1.30-1.85	0.10 (0.07, 0.13)	0.14 (0.11, 0.16)	0.14 (0.10, 0.18)	0.12 (0.10, 0.15)	0.15 (0.11, 0.19)	0.12 (0.08, 0.17)
1.85-3,00	0.12 (0.08, 0.17)	0.09 (0.07, 0.12)	0.09 (0.06, 0.12)	0.10 (0.08, 0.13)	0.10 (0.06, 0.14)	0.08 (0.05, 0.12)
>=3.00	0.06 (0.04, 0.09)	0.04 (0.03, 0.06)	0.06 (0.03, 0.08)	0.05 (0.03, 0.07)	0.06 (0.03, 0.10)	0.06 (0.00, 0.12)
Survey wave						
2007-2008	0.12 (0.08, 0.17)	0.11 (0.08, 0.15)	0.12 (0.08, 0.15)	0.13 (0.09, 0.17)	0.10 (0.07, 0.13)	0.10 (0.07, 0.14)

© 2025 Li Q & Zhao S. JAMA Health Forum.

2009-2010	0.15 (0.10, 0.20)	0.17 (0.13, 0.21)	0.14 (0.11, 0.17)	0.15 (0.12, 0.18)	0.17 (0.12, 0.22)	0.13 (0.09, 0.16)
2011-2012	0.20 (0.15, 0.25)	0.19 (0.14, 0.24)	0.18 (0.12, 0.24)	0.18 (0.14, 0.23)	0.19 (0.13, 0.25)	0.19 (0.13, 0.26)
2013-2014	0.22 (0.14, 0.29)	0.18 (0.14, 0.22)	0.19 (0.16, 0.22)	0.21 (0.16, 0.26)	0.16 (0.11, 0.21)	0.18 (0.13, 0.23)
2015-2016	0.15 (0.11, 0.20)	0.18 (0.13, 0.23)	0.19 (0.15, 0.23)	0.17 (0.13, 0.21)	0.20 (0.15, 0.25)	0.19 (0.13, 0.25)
2017-2018	0.16 (0.12, 0.19)	0.17 (0.14, 0.19)	0.19 (0.15, 0.23)	0.17 (0.13, 0.20)	0.18 (0.15, 0.22)	0.21 (0.16, 0.25)

Abbreviations: HEI, Healthy Eating Index; NHANES, National Health and Nutrition Examination Survey.

[a] Mean values were adjusted for NHANES dietary weights. These weights account for the complex survey design, including oversampling, survey nonresponse, and poststratification.

[b] Obesity status was categorized according to the World Health Organization (WHO) definitions: normal (BMI<25), overweight (BMI ≥ 25) and obesity (BMI ≥ 30)

[c] Diabetes status was categorized as follows: No diabetes (<5.7%), Pre-diabetes (5.7% to 6.4%), and Diabetes (≥6.5%)

[d] Other store types include combination, specialty, and miscellaneous stores.

[e] “Other” includes race/ethnicity other than non-Hispanic white, non-Hispanic black, and Hispanic, including multiracial.

[f] Represents the ratio of family income to the federal poverty threshold, adjusted for household size. Higher ratios indicate higher income levels.

© 2025 Li Q & Zhao S. JAMA Health Forum.

eTable 3. Store Availability and Average Minimum Distance to SNAP-Authorized Stores Among Adult SNAP Participants in the NHANES

	Weighted Mean (95% CI)[a]					
Store Availability	Any Store Type	Grocery	Supermarket	Superstore	Convenience	Other[b]
Average Minimum Distance, mile	0.62 (0.53, 0.71)	2.24 (1.90, 2.58)	2.39 (1.95, 2.83)	2.71 (2.32, 3.10)	0.87 (0.77, 0.97)	1.34 (1.00, 1.69)
Less than 0.1 mile	0.15 (0.12, 0.17)	0.05 (0.04, 0.07)	0.01 (0.00, 0.02)	0.02 (0.01, 0.02)	0.09 (0.08, 0.11)	0.03 (0.01, 0.04)
0.1 to 0.25 miles	0.29 (0.26, 0.32)	0.12 (0.09, 0.15)	0.05 (0.03, 0.06)	0.06 (0.04, 0.08)	0.23 (0.20, 0.26)	0.16 (0.14, 0.18)
0.25 to 0.5 miles	0.26 (0.23, 0.29)	0.14 (0.11, 0.17)	0.13 (0.11, 0.15)	0.12 (0.10, 0.14)	0.26 (0.24, 0.29)	0.25 (0.21, 0.28)
0.5 to 1 mile	0.16 (0.14, 0.18)	0.18 (0.15, 0.20)	0.21 (0.17, 0.25)	0.26 (0.22, 0.29)	0.21 (0.18, 0.24)	0.29 (0.26, 0.32)
Greater than 1 mile	0.14 (0.11, 0.18)	0.51 (0.45, 0.57)	0.60 (0.56, 0.64)	0.55 (0.50, 0.60)	0.20 (0.18, 0.23)	0.28 (0.24, 0.32)

Abbreviations: NHANES, National Health and Nutrition Examination Survey.
[a] Mean values were adjusted for NHANES dietary weights. These weights account for the complex survey design, including oversampling, survey nonresponse, and poststratification.
[b] Other store types include combination, specialty, and miscellaneous stores.

© 2025 Li Q & Zhao S. JAMA Health Forum.

eFigure 1. Unadjusted Estimated HEI-2015 Difference by Availability of SNAP-Authorized Store Types Among SNAP Recipients, Relative to Living More Than 1 Mile Away

Store Available	Coefficient (95% CI)
**Any**	
<= 0.1 Miles	3.14 [1.12; 5.15]
>0.1 to <=0.25 Miles	3.15 [1.33; 4.97]
>0.25 to <=0.5 Miles	1.82 [0.62; 3.02]
>0.5 to <=1 Miles	1.49 [-0.05; 3.03]
**Grocery**	
<= 0.1 Miles	4.68 [2.49; 6.87]
>0.1 to <=0.25 Miles	3.54 [1.66; 5.43]
>0.25 to <=0.5 Miles	0.72 [-0.81; 2.26]
>0.5 to <=1 Miles	1.67 [-0.01; 3.35]
**Supermarket**	
<= 0.1 Miles	-1.65 [-5.93; 2.63]
>0.1 to <=0.25 Miles	3.88 [0.91; 6.86]
>0.25 to <=0.5 Miles	2.61 [0.45; 4.76]
>0.5 to <=1 Miles	1.88 [0.02; 3.73]
**Superstore**	
<= 0.1 Miles	7.41 [3.41; 11.42]
>0.1 to <=0.25 Miles	5.27 [1.91; 8.63]
>0.25 to <=0.5 Miles	1.59 [-1.22; 4.40]
>0.5 to <=1 Miles	1.88 [0.31; 3.46]
**Convenience**	
<= 0.1 Miles	1.83 [-0.28; 3.93]
>0.1 to <=0.25 Miles	2.28 [0.85; 3.71]
>0.25 to <=0.5 Miles	1.18 [0.03; 2.33]
>0.5 to <=1 Miles	1.19 [0.06; 2.32]
**Other**	
<= 0.1 Miles	2.55 [-1.61; 6.71]
>0.1 to <=0.25 Miles	2.83 [0.85; 4.81]
>0.25 to <=0.5 Miles	1.55 [0.20; 2.90]
>0.5 to <=1 Miles	1.02 [-0.43; 2.46]

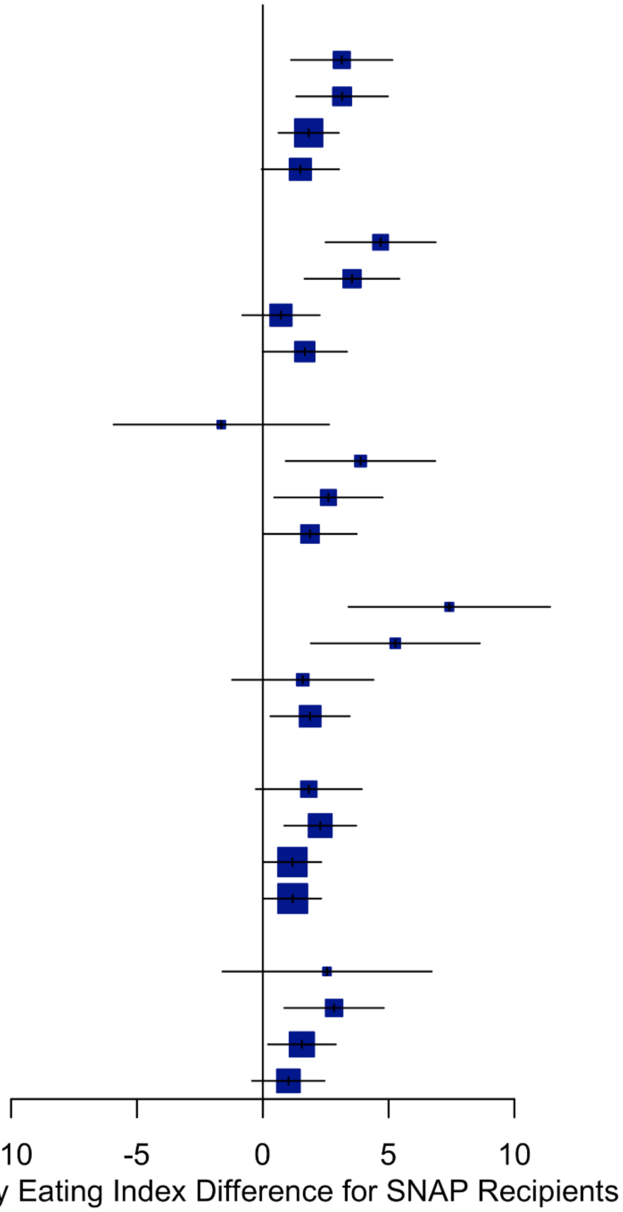

Squares represent the estimated HEI-2015 differences for SNAP recipients by availability of various SNAP-authorized store types within specified distances relative to being more than 1 mile away, with horizontal lines indicating the 95% CIs. The store types analyzed include grocery stores, supermarkets, superstores, and convenience stores, as well as a category for all store types combined. Positive coefficients indicate higher HEI-2015 scores for closer proximity to the respective store type compared to being farther than 1 mile away. Exact coefficient values and CIs are provided in the table to the left of the plot.

© 2025 Li Q & Zhao S. JAMA Health Forum.

eFigure 2. Unadjusted Estimated HEI-2015 Difference by Availability of SNAP-Authorized Store Types Among SNAP Recipients by Weight Status, Relative to Living More Than 1 Mile Away

Panel A: Normal Weight (BMI < 25)

Store Available	Coefficient (95% CI)
**Any**	
<= 0.1 Miles	4.50 [0.87; 8.14]
>0.1 to <=0.25 Miles	4.87 [1.64; 8.11]
>0.25 to <=0.5 Miles	2.50 [-1.10; 6.09]
>0.5 to <=1 Miles	1.50 [-2.16; 5.16]
**Grocery**	
<= 0.1 Miles	3.42 [0.38; 6.46]
>0.1 to <=0.25 Miles	4.20 [1.46; 6.94]
>0.25 to <=0.5 Miles	-0.00 [-2.56; 2.56]
>0.5 to <=1 Miles	-0.19 [-3.38; 3.01]
**Supermarket**	
<= 0.1 Miles	0.40 [-3.28; 4.08]
>0.1 to <=0.25 Miles	4.43 [1.06; 7.80]
>0.25 to <=0.5 Miles	2.98 [-1.50; 7.47]
>0.5 to <=1 Miles	3.10 [0.30; 5.90]
**Superstore**	
<= 0.1 Miles	5.68 [1.52; 9.85]
>0.1 to <=0.25 Miles	4.99 [0.54; 9.44]
>0.25 to <=0.5 Miles	2.60 [-1.87; 7.08]
>0.5 to <=1 Miles	1.23 [-0.98; 3.43]
**Convenience**	
<= 0.1 Miles	3.10 [-1.19; 7.39]
>0.1 to <=0.25 Miles	3.13 [1.11; 5.15]
>0.25 to <=0.5 Miles	0.38 [-2.38; 3.15]
>0.5 to <=1 Miles	1.32 [-1.18; 3.82]
**Other**	
<= 0.1 Miles	1.15 [-3.37; 5.68]
>0.1 to <=0.25 Miles	2.88 [0.12; 5.64]
>0.25 to <=0.5 Miles	0.62 [-1.62; 2.86]
>0.5 to <=1 Miles	0.56 [-1.86; 2.97]

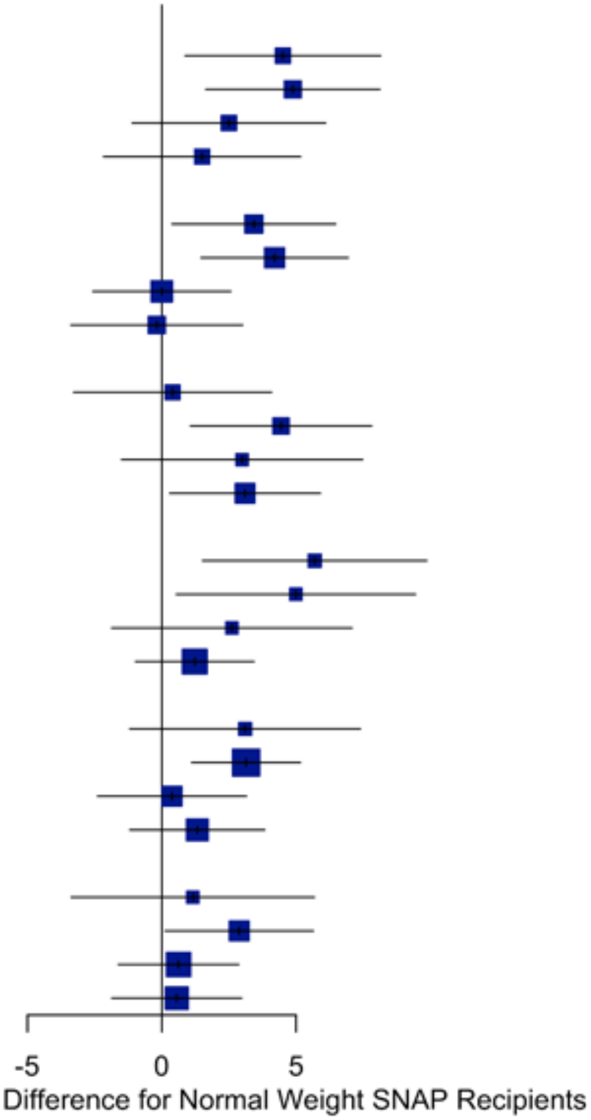

Squares represent the estimated HEI-2015 differences for SNAP recipients by availability of SNAP-authorized store types at specified distances, with 95% Cis shown as horizontal lines.

© 2025 Li Q & Zhao S. JAMA Health Forum.

eFigure 2. Unadjusted Estimated HEI-2015 Difference by Availability of SNAP-Authorized Store Types Among SNAP Recipients by Weight Status, Relative to Living More Than 1 Mile Away

Panel B: Overweight (BMI ≥ 25)

Store Available	Coefficient (95% CI)
**Any**	
<= 0.1 Miles	2.93 [-0.90; 6.76]
>0.1 to <=0.25 Miles	2.72 [-0.92; 6.35]
>0.25 to <=0.5 Miles	2.63 [-0.38; 5.64]
>0.5 to <=1 Miles	1.46 [-2.64; 5.56]
**Grocery**	
<= 0.1 Miles	6.04 [1.26; 10.81]
>0.1 to <=0.25 Miles	3.44 [-0.38; 7.26]
>0.25 to <=0.5 Miles	0.45 [-1.65; 2.54]
>0.5 to <=1 Miles	-0.26 [-2.72; 2.20]
**Supermarket**	
<= 0.1 Miles	-3.46 [-8.01; 1.09]
>0.1 to <=0.25 Miles	3.77 [-1.79; 9.32]
>0.25 to <=0.5 Miles	3.49 [0.26; 6.72]
>0.5 to <=1 Miles	-0.04 [-2.98; 2.90]
**Superstore**	
<= 0.1 Miles	2.71 [-4.10; 9.53]
>0.1 to <=0.25 Miles	3.70 [-0.85; 8.24]
>0.25 to <=0.5 Miles	2.38 [-1.81; 6.58]
>0.5 to <=1 Miles	1.43 [-1.19; 4.05]
**Convenience**	
<= 0.1 Miles	0.36 [-3.66; 4.38]
>0.1 to <=0.25 Miles	1.05 [-2.62; 4.73]
>0.25 to <=0.5 Miles	1.27 [-1.78; 4.33]
>0.5 to <=1 Miles	-0.52 [-4.05; 3.02]
**Other**	
<= 0.1 Miles	2.85 [-0.75; 6.46]
>0.1 to <=0.25 Miles	4.49 [1.25; 7.72]
>0.25 to <=0.5 Miles	2.28 [0.03; 4.53]
>0.5 to <=1 Miles	1.53 [-1.71; 4.77]

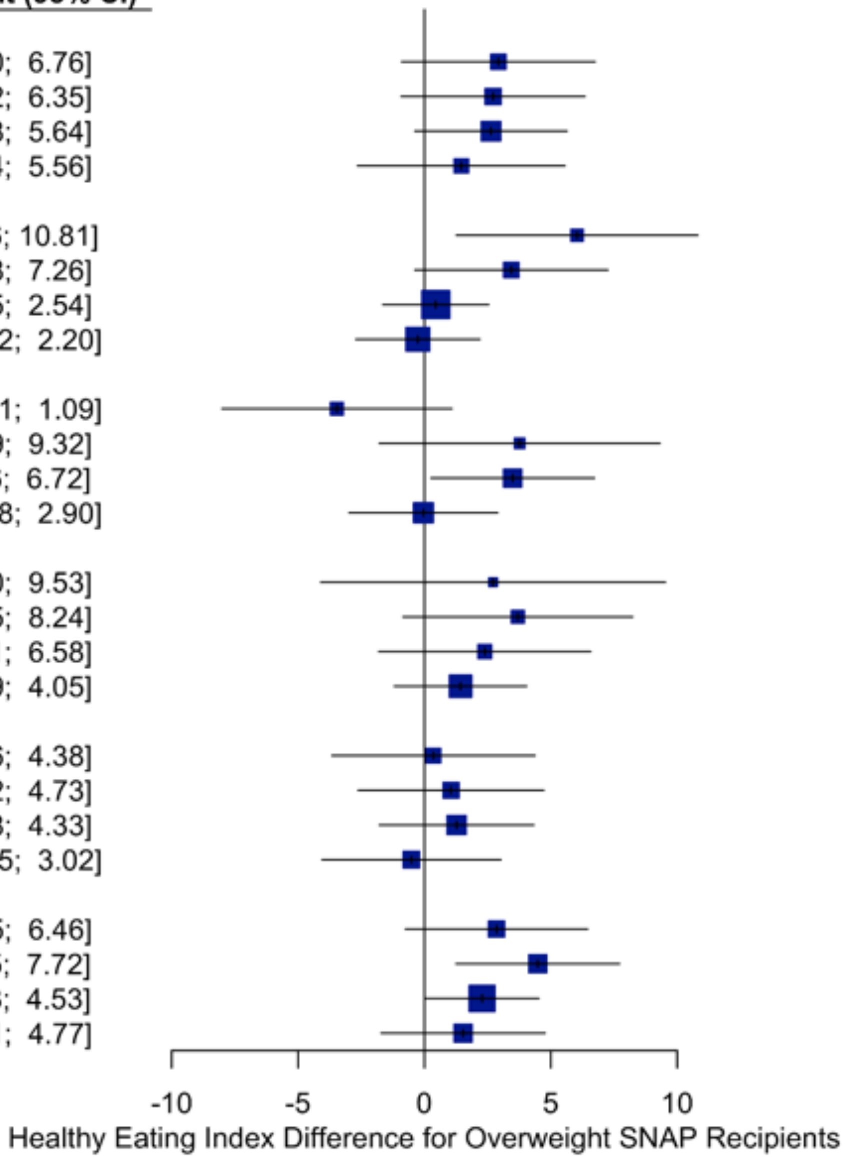

Squares represent the estimated HEI-2015 differences for SNAP recipients by availability of SNAP-authorized store types at specified distances, with 95% Cis shown as horizontal lines.

© 2025 Li Q & Zhao S. JAMA Health Forum.

eFigure 2. Unadjusted Estimated HEI-2015 Difference by Availability of SNAP-Authorized Store Types Among SNAP Recipients by Weight Status, Relative to Living More Than 1 Mile Away

Panel C: Obese (BMI ≥ 30)

Store Available	Coefficient (95% CI)
**Any**	
<= 0.1 Miles	2.61 [-0.32; 5.54]
>0.1 to <=0.25 Miles	2.38 [0.34; 4.42]
>0.25 to <=0.5 Miles	0.79 [-1.16; 2.75]
>0.5 to <=1 Miles	1.46 [-1.07; 4.00]
**Grocery**	
<= 0.1 Miles	4.39 [0.21; 8.57]
>0.1 to <=0.25 Miles	3.30 [0.33; 6.28]
>0.25 to <=0.5 Miles	1.22 [-0.60; 3.03]
>0.5 to <=1 Miles	3.97 [2.06; 5.88]
**Supermarket**	
<= 0.1 Miles	-1.65 [-8.69; 5.39]
>0.1 to <=0.25 Miles	3.50 [-0.35; 7.34]
>0.25 to <=0.5 Miles	1.46 [-0.63; 3.54]
>0.5 to <=1 Miles	2.51 [0.20; 4.82]
**Superstore**	
<= 0.1 Miles	10.26 [5.27; 15.24]
>0.1 to <=0.25 Miles	6.49 [1.68; 11.30]
>0.25 to <=0.5 Miles	0.74 [-1.70; 3.19]
>0.5 to <=1 Miles	2.58 [0.64; 4.51]
**Convenience**	
<= 0.1 Miles	2.10 [-0.62; 4.82]
>0.1 to <=0.25 Miles	2.51 [0.73; 4.28]
>0.25 to <=0.5 Miles	1.30 [-0.58; 3.18]
>0.5 to <=1 Miles	2.10 [0.06; 4.14]
**Other**	
<= 0.1 Miles	3.02 [-3.07; 9.11]
>0.1 to <=0.25 Miles	1.90 [-0.62; 4.42]
>0.25 to <=0.5 Miles	1.64 [0.06; 3.22]
>0.5 to <=1 Miles	0.65 [-1.10; 2.39]

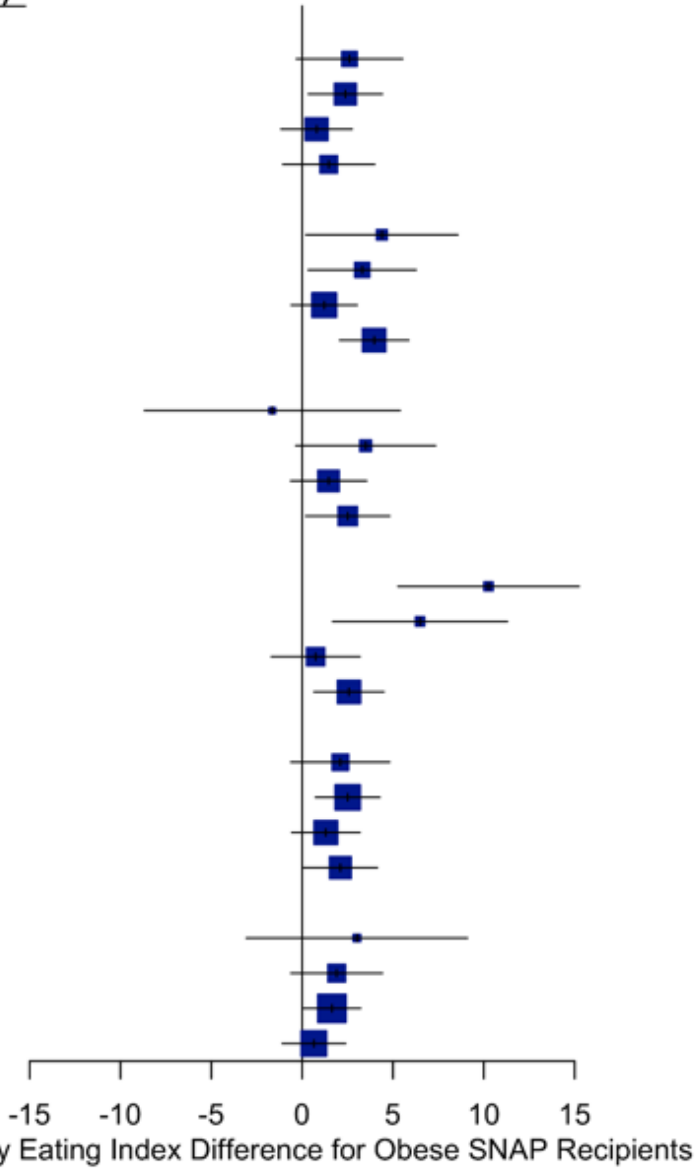

Squares represent the estimated HEI-2015 differences for SNAP recipients by availability of SNAP-authorized store types at specified distances, with 95% Cis shown as horizontal lines.

© 2025 Li Q & Zhao S. JAMA Health Forum.

eFigure 3. Unadjusted Estimated HEI-2015 Difference by Availability of SNAP-Authorized Store Types Among SNAP Recipients by Diabetes Status, Relative to Living More Than 1 Mile Away

Panel A: No Diabetes (HbA1c < 5.7%)

Store Available	Coefficient (95% CI)
**Any**	
<= 0.1 Miles	2.39 [0.20; 4.57]
>0.1 to <=0.25 Miles	2.38 [0.43; 4.34]
>0.25 to <=0.5 Miles	1.41 [-0.32; 3.15]
>0.5 to <=1 Miles	1.27 [-1.09; 3.63]
**Grocery**	
<= 0.1 Miles	3.85 [1.24; 6.47]
>0.1 to <=0.25 Miles	3.19 [0.57; 5.80]
>0.25 to <=0.5 Miles	0.32 [-1.30; 1.93]
>0.5 to <=1 Miles	0.84 [-1.10; 2.79]
**Supermarket**	
<= 0.1 Miles	-2.52 [-7.27; 2.22]
>0.1 to <=0.25 Miles	4.48 [1.17; 7.78]
>0.25 to <=0.5 Miles	2.56 [-0.58; 5.69]
>0.5 to <=1 Miles	1.75 [-0.28; 3.77]
**Superstore**	
<= 0.1 Miles	8.22 [2.23; 14.20]
>0.1 to <=0.25 Miles	4.33 [0.91; 7.76]
>0.25 to <=0.5 Miles	1.41 [-2.13; 4.95]
>0.5 to <=1 Miles	1.90 [-0.23; 4.03]
**Convenience**	
<= 0.1 Miles	0.41 [-2.25; 3.07]
>0.1 to <=0.25 Miles	1.83 [0.28; 3.38]
>0.25 to <=0.5 Miles	0.36 [-1.40; 2.13]
>0.5 to <=1 Miles	0.58 [-1.22; 2.38]
**Other**	
<= 0.1 Miles	3.22 [-2.07; 8.51]
>0.1 to <=0.25 Miles	2.19 [-0.29; 4.66]
>0.25 to <=0.5 Miles	1.78 [0.06; 3.51]
>0.5 to <=1 Miles	1.16 [-0.49; 2.81]

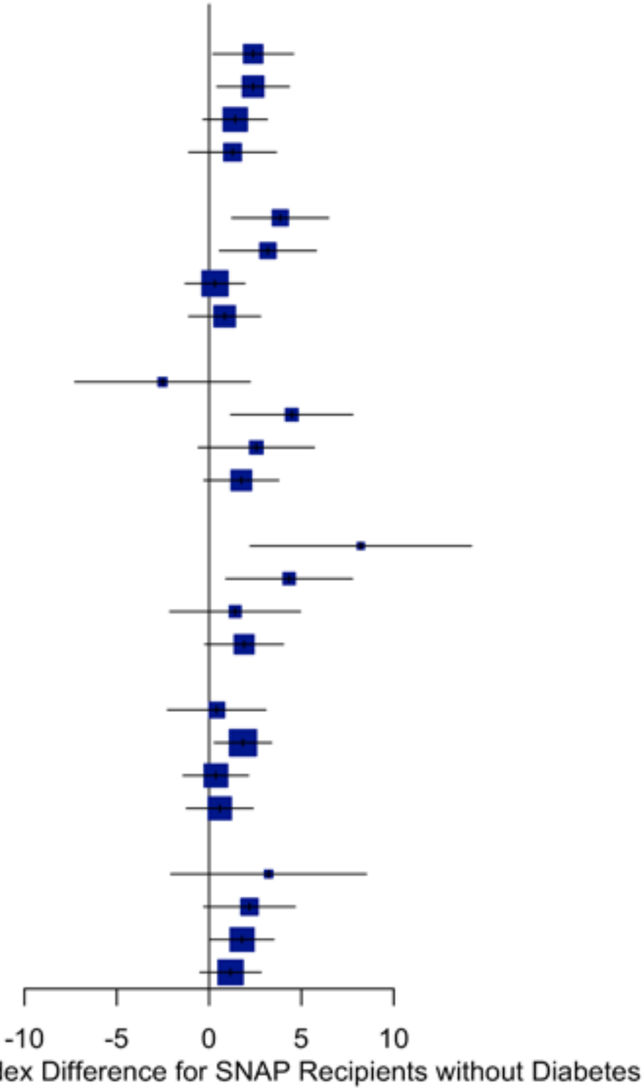

Squares represent the estimated HEI-2015 differences for SNAP recipients by availability of SNAP-authorized store types at specified distances, with 95% Cis shown as horizontal lines.

© 2025 Li Q & Zhao S. JAMA Health Forum.

eFigure 3. Unadjusted Estimated HEI-2015 Difference by Availability of SNAP-Authorized Store Types Among SNAP Recipients by Diabetes Status, Relative to Living More Than 1 Mile Away

Panel B: Prediabetes (HbA1c 5.7% to 6.4%)

Store Available	Coefficient (95% CI)
**Any**	
<= 0.1 Miles	3.57 [-1.07; 8.20]
>0.1 to <=0.25 Miles	4.36 [-0.21; 8.92]
>0.25 to <=0.5 Miles	1.62 [-2.40; 5.64]
>0.5 to <=1 Miles	0.24 [-3.98; 4.46]
**Grocery**	
<= 0.1 Miles	6.40 [2.03; 10.78]
>0.1 to <=0.25 Miles	4.32 [1.22; 7.41]
>0.25 to <=0.5 Miles	1.93 [-1.13; 4.99]
>0.5 to <=1 Miles	2.94 [-0.23; 6.10]
**Supermarket**	
<= 0.1 Miles	-1.01 [-6.21; 4.18]
>0.1 to <=0.25 Miles	4.46 [1.23; 7.68]
>0.25 to <=0.5 Miles	3.38 [0.09; 6.68]
>0.5 to <=1 Miles	2.57 [0.28; 4.85]
**Superstore**	
<= 0.1 Miles	6.49 [-2.10; 15.08]
>0.1 to <=0.25 Miles	6.75 [1.17; 12.33]
>0.25 to <=0.5 Miles	2.40 [-1.21; 6.01]
>0.5 to <=1 Miles	1.81 [-0.80; 4.43]
**Convenience**	
<= 0.1 Miles	3.92 [-0.52; 8.36]
>0.1 to <=0.25 Miles	3.68 [-0.38; 7.75]
>0.25 to <=0.5 Miles	2.96 [-0.34; 6.26]
>0.5 to <=1 Miles	1.41 [-2.63; 5.46]
**Other**	
<= 0.1 Miles	-0.81 [-4.46; 2.83]
>0.1 to <=0.25 Miles	4.22 [0.83; 7.61]
>0.25 to <=0.5 Miles	0.51 [-3.53; 4.55]
>0.5 to <=1 Miles	0.55 [-2.80; 3.90]

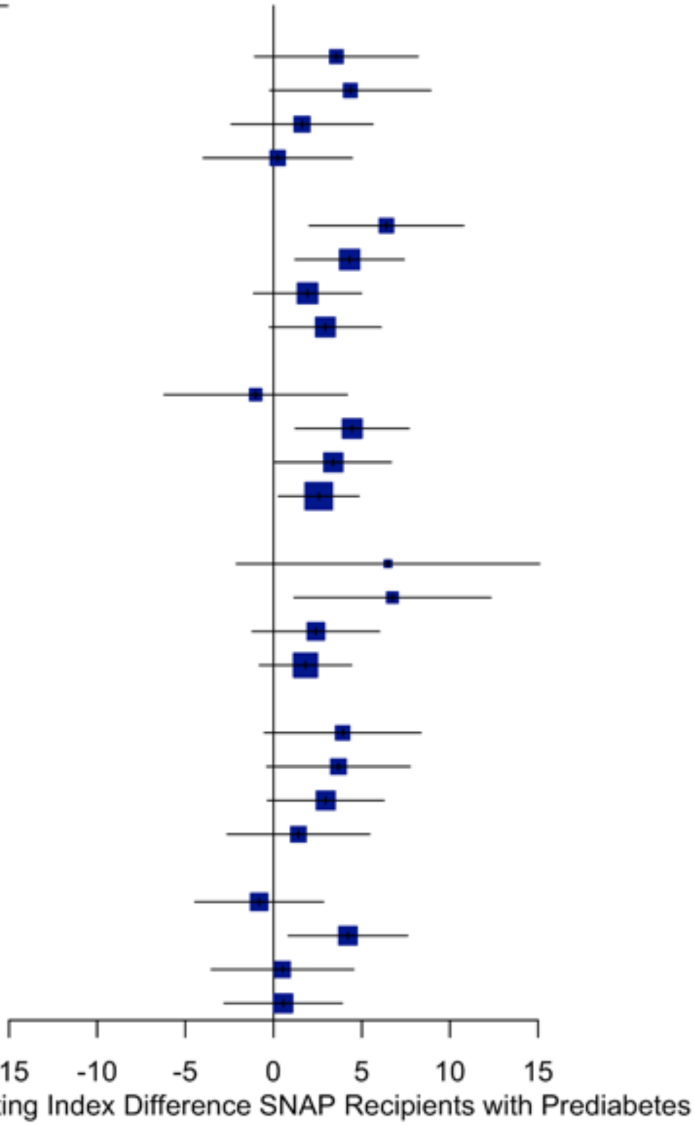

Squares represent the estimated HEI-2015 differences for SNAP recipients by availability of SNAP-authorized store types at specified distances, with 95% Cis shown as horizontal lines.

© 2025 Li Q & Zhao S. JAMA Health Forum.

eFigure 3. Unadjusted Estimated HEI-2015 Difference by Availability of SNAP-Authorized Store Types Among SNAP Recipients by Diabetes Status, Relative to Living More Than 1 Mile Away

Panel C: Diabetes (HbA1c ≥ 6.4%)

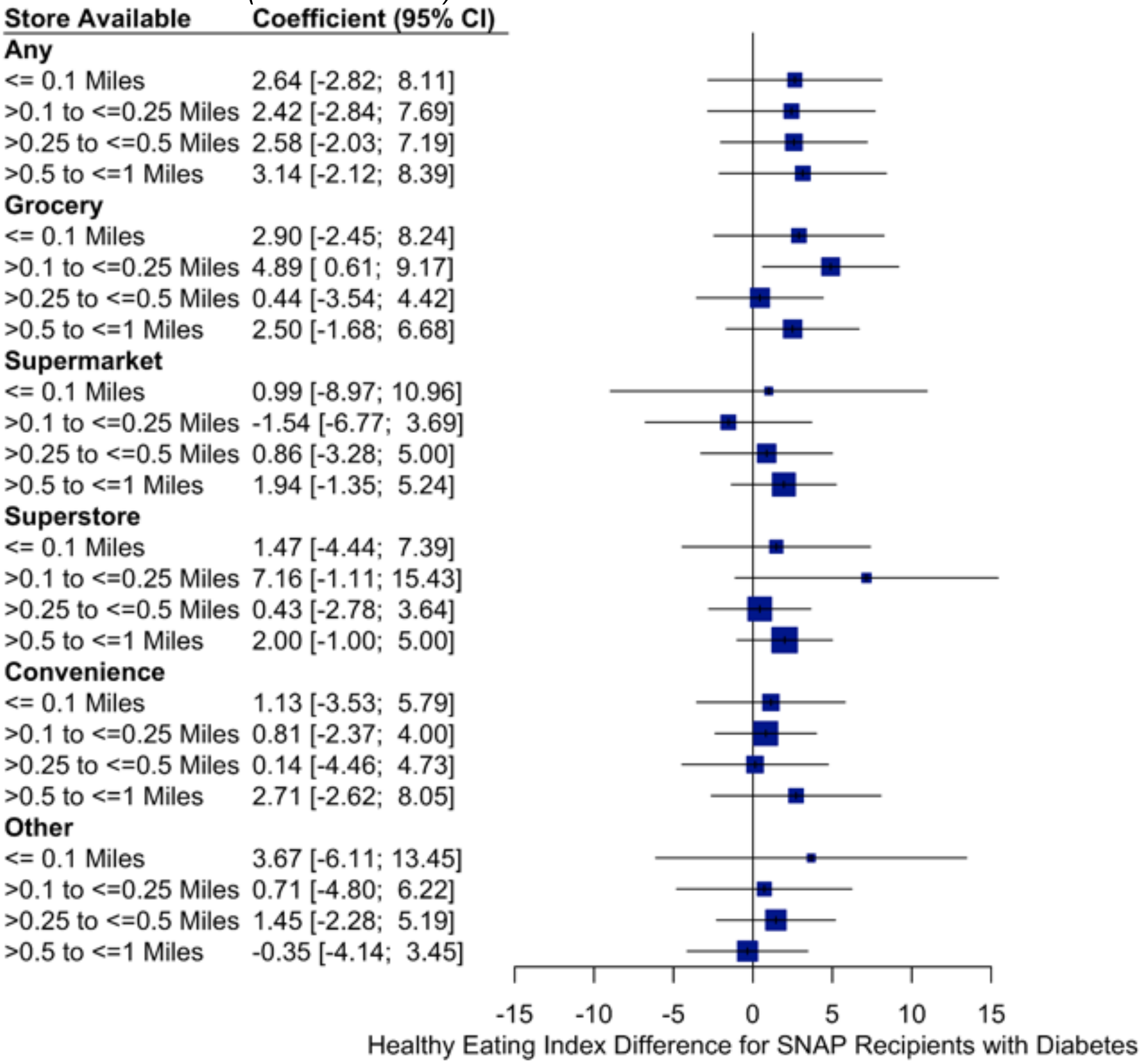

Squares represent the estimated HEI-2015 differences for SNAP recipients by availability of SNAP-authorized store types at specified distances, with 95% Cis shown as horizontal lines

© 2025 Li Q & Zhao S. JAMA Health Forum.

eFigure 4. Unadjusted Estimated HEI-2015 Difference by Distance to SNAP-Authorized Store Types, by Obesity and Diabetes Status

Each Additional Mile	Coefficient (95% CI)
**All**	
Any	-0.92 [-1.46; -0.38]
Grocery	-0.30 [-0.53; -0.07]
Supermarket	-0.22 [-0.34; -0.10]
Superstore	-0.15 [-0.28; -0.03]
Convenience	-0.36 [-0.61; -0.12]
Other	-0.33 [-0.49; -0.18]
**Obese**	
Any	-0.90 [-1.51; -0.28]
Grocery	-0.30 [-0.59; -0.02]
Supermarket	-0.22 [-0.43; -0.00]
Superstore	-0.13 [-0.33; 0.08]
Convenience	-0.44 [-0.78; -0.09]
Other	-0.35 [-0.55; -0.15]
**Overweight**	
Any	-0.81 [-1.62; -0.01]
Grocery	-0.47 [-0.75; -0.20]
Supermarket	-0.21 [-0.38; -0.04]
Superstore	-0.29 [-0.48; -0.11]
Convenience	-0.13 [-0.61; 0.35]
Other	-0.32 [-0.53; -0.11]
**Normal weight**	
Any	-1.05 [-1.91; -0.19]
Grocery	-0.11 [-0.38; 0.16]
Supermarket	-0.30 [-0.47; -0.13]
Superstore	-0.04 [-0.26; 0.18]
Convenience	-0.51 [-1.13; 0.12]
Other	-0.35 [-0.83; 0.13]
**Diabetes**	
Any	-1.36 [-2.53; -0.20]
Grocery	-0.27 [-0.87; 0.34]
Supermarket	-0.18 [-0.62; 0.26]
Superstore	-0.10 [-0.46; 0.27]
Convenience	-0.52 [-1.07; 0.03]
Other	-0.06 [-0.45; 0.33]
**Prediabetes**	
Any	-1.21 [-2.17; -0.25]
Grocery	-0.24 [-0.63; 0.15]
Supermarket	-0.32 [-0.53; -0.11]
Superstore	-0.22 [-0.56; 0.13]
Convenience	-0.97 [-1.68; -0.26]
Other	-0.47 [-0.81; -0.14]
**No Diabetes**	
Any	-0.71 [-1.30; -0.11]
Grocery	-0.36 [-0.54; -0.19]
Supermarket	-0.19 [-0.33; -0.06]
Superstore	-0.15 [-0.26; -0.04]
Convenience	-0.17 [-0.53; 0.20]
Other	-0.31 [-0.49; -0.14]

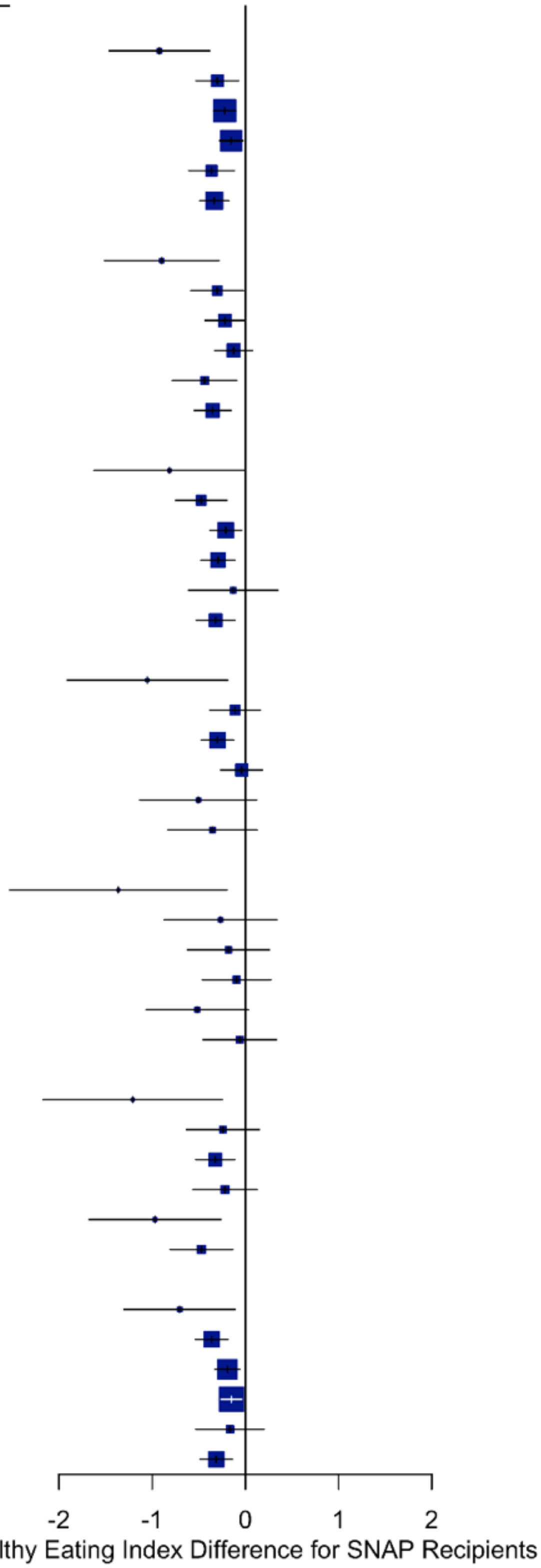

© 2025 Li Q & Zhao S. JAMA Health Forum.

eFigure 5. Estimated HEI-2015 Component Differences per Additional Mile of Distance to SNAP-Authorized Stores Among SNAP Recipients

Each Additional Mile	Coefficient (95% CI)
**Per mile**	
Total vegetables	-0.07 [-0.15; 0.02]
Greens and beans	-0.10 [-0.16; -0.03]
Total fruit	-0.15 [-0.20; -0.10]
Whole fruit	-0.14 [-0.20; -0.09]
Whole grains	-0.04 [-0.13; 0.04]
Total Dairy	0.04 [-0.11; 0.18]
Total protein food	-0.04 [-0.10; 0.02]
Seafood and plant proteins	-0.08 [-0.16; 0.00]
Fatty acids	-0.09 [-0.23; 0.05]
Sodium	0.02 [-0.07; 0.10]
Refined grains	0.07 [-0.06; 0.20]
Saturated fats	-0.10 [-0.25; 0.04]
Added sugars	-0.16 [-0.33; 0.01]

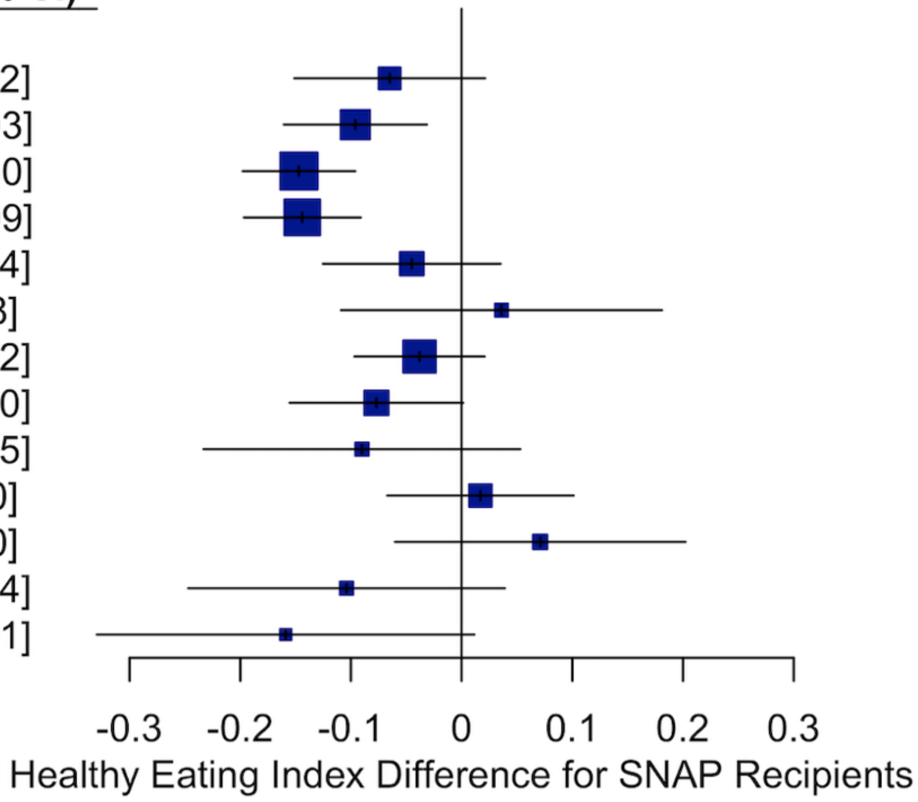

© 2025 Li Q & Zhao S. JAMA Health Forum.

eFigure 6. Unadjusted Estimated HEI-2015 Component Differences per Additional Mile of Distance to SNAP-Authorized Stores Among SNAP Recipients

Each Additional Mile	Coefficient (95% CI)
**Per mile**	
Total vegetables	-0.05 [-0.14; 0.04]
Greens and beans	-0.09 [-0.15; -0.03]
Total fruit	-0.15 [-0.20; -0.10]
Whole fruit	-0.13 [-0.18; -0.07]
Whole grains	-0.04 [-0.12; 0.03]
Total Dairy	0.10 [-0.03; 0.23]
Total protein food	-0.05 [-0.10; 0.01]
Seafood and plant proteins	-0.08 [-0.14; -0.01]
Fatty acids	-0.14 [-0.29; 0.00]
Sodium	0.03 [-0.05; 0.10]
Refined grains	0.03 [-0.10; 0.17]
Saturated fats	-0.11 [-0.24; 0.03]
Added sugars	-0.16 [-0.35; 0.03]

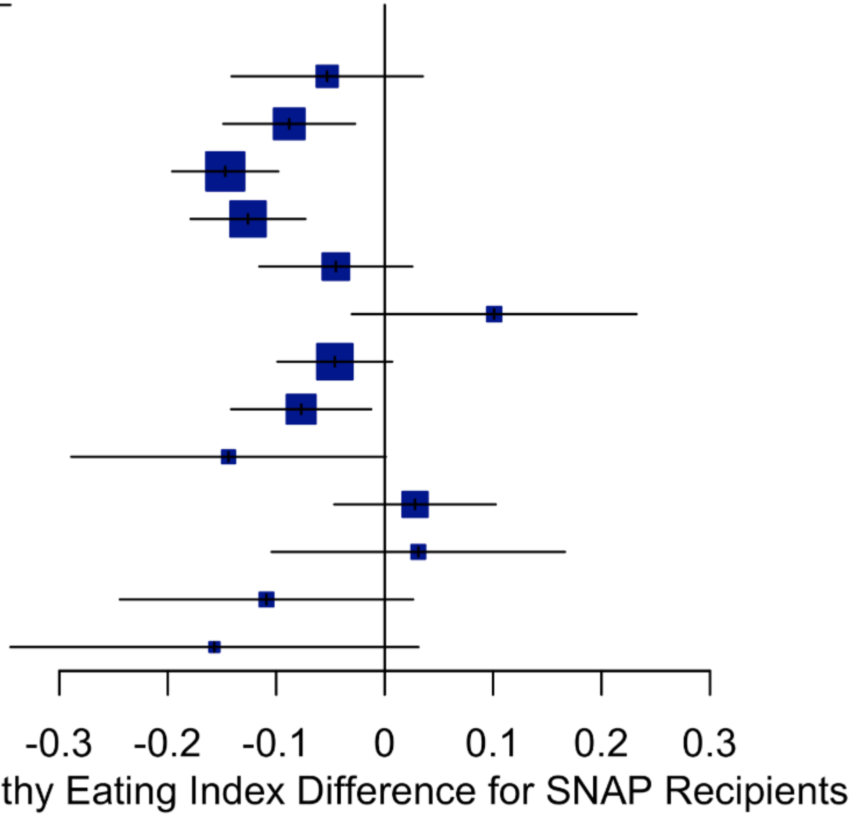

© 2025 Li Q & Zhao S. JAMA Health Forum.
