# Supplementary material for: Access to SNAP-Authorized Retailers and Diet Quality Among SNAP Recipients
Source: JAMA Health Forum. 2025 Apr 18;6(4):e250677. doi: 10.1001/jamahealthforum.2025.0677 (PMC12008766; doi:10.1001/jamahealthforum.2025.0677)
Supplement: Supplement 2. — Data Sharing Statement [file jamahealthforum-e250677-s002.pdf]

Data Sharing Statement

Li. Access to SNAP-Authorized Retailers and Diet Quality Among SNAP Recipients. *JAMA Health Forum*. Published April 18, 2025. doi:10.1001/jamahealthforum.2025.0677

Data
Data available: No

Additional Information
Explanation for why data not available: Restricted-use National Health and Nutrition Examination Survey (NHANES) data is not available for public sharing due to data privacy and confidentiality requirements. However, we can provide the code used for our analysis upon request.
